# Supplementary material for: MERGE-RNA: a physics-based model to predict RNA secondary structure ensembles with chemical probing
Source: Nucleic Acids Res. 2026 Sep 22;54(18):gkag917. doi: 10.1093/nar/gkag917 (PMC13594980; doi:10.1093/nar/gkag917)
Supplement: Supplementary file 1 [file gkag917_supplemental_file.pdf]

Supplementary Information

Physical Model

The physical model underlying our analysis of RNA chemical probing experiments is illustrated in the left panel of Figure 1 and will be detailed in this section. We derive and justify the expression for the free energy of a secondary structure s in the presence of a chemical probe (as reported in the main text) which enables the prediction of the ensemble of structures, and explain our methodology for producing a prediction of the reactivity profiles at varying probe concentrations, which serve as the theoretical basis for fitting experimental data.

Secondary structure populations

The initial step in our analysis involves modeling the RNA folding into an ensemble of secondary structures, while also accounting for interactions with chemical probes. Our baseline is the prediction of the ViennaRNA package (20), specifically employing the `RNA.fold_compound.subopt` method to obtain an ensemble of suboptimal structures s with their associated free energies $F_0(s)$.

The maximum entropy formalism (21) provides a principled framework for integrating experimental data while making minimal adjustments to the baseline predictions from the classical thermodynamic model, ensuring that the resulting distribution of observables gets consistent with experimental measurements. Within this framework, the minimal perturbation to the baseline model that aligns with experimental data is achieved by introducing sequence-specific model parameters λ_i –one for each nucleotide position– that serve as Lagrange multipliers constraining the ensemble to match experimentally observed properties. These soft constraints can be effectively interpreted as positional corrections of pairing energy, allowing us to capture sequence-dependent effects that may not be fully accounted for in the standard thermodynamic model. This formalism allows us to seamlessly integrate experimental data into the thermodynamic framework, and the corrections will be applied only where there are discrepancies between the model and the data, while leaving the areas where there is agreement unchanged. Importantly, the λ_i parameters are sequence-specific but remain consistent across all concentrations and experimental replicates for the same sequence.

The population of each secondary structure s is determined by its free energy, which comprises three components: the baseline free energy $E_0(s)$, sequence-specific soft constraints λ_i, and the contribution from probe interactions $\Delta F_{\text{[DMS]}}(s)$:

$$F(s) = F_0(s) + \sum_{i \in \text{paired}(s)} \lambda_i + \Delta F_{\text{[DMS]}}(s) \tag{7}$$

The last term explicitly accounts for interactions between chemical probes and RNA structures, which modify the free energy in a concentration-dependent manner, whose explicit form is derived below. The concentration-dependent energy of the probe-RNA interaction is described by chemical potential μ. Note that once a chemical potential for a reference probe concentration is known, the chemical potential for any other concentration can be derived from the ratio of the experimental concentrations as $\mu = \mu_r + \mathrm{k_B T} \ln\left(\frac{\text{[DMS]}}{\text{[DMS]}_r}\right)$, where [DMS] is the concentration of the probe in the experiment and [DMS]_r and μ_r are respectively the reference concentration and its chemical potential. Since for most chemical probes the interaction energy depends on local RNA structure (this is especially true for the most widely used SHAPE and DMS), we include an additional parameter $\Delta\mu_{\text{pairing}}$ that represents the energetic penalty for probe interactions with paired nucleotides, such that the effective binding energy for paired nucleotides becomes $\mu' \equiv \mu - \Delta\mu_{\text{pairing}}$. This formulation allows us to model the effect of varying probe concentrations with only two parameters (μ_r and $\Delta\mu_{\text{pairing}}$) and thus to combine data from multiple experiments into a single, coherent framework.

Importantly, in what follows we consider only the binding partition function, i.e., the contribution to the total partition function arising from independent probe-RNA binding degrees of freedom at fixed secondary structure s. Under the assumption of independent binding across sites, this factorizes as

$$Z_{\text{bind}}(s) = \prod_{i:\, s_i=0} z_u \prod_{i:\, s_i=1} z_p \tag{8}$$

$$= z_u^{N_u(s)}\, z_p^{N_p(s)} \tag{9}$$

with single-site partition functions $z_u = 1 + \mathrm{e}^{\beta\mu}$ for unpaired sites and $z_p = 1 + \mathrm{e}^{\beta\mu'}$ for paired sites, and where $N_u(s)$ and $N_p(s)$ denote the numbers of unpaired and paired nucleotides in structure s. The corresponding concentration-dependent contribution to the free energy (the last term in Equation 7) is then

$$\beta\Delta F_{\text{[DMS]}}(s) = -\ln\left(Z_{\text{bind}}(s)\right) \tag{10}$$

$$= -N_u(s)\ln z_u - N_p(s)\ln z_p \tag{11}$$

$$= -N_u(s)\ln(1+\mathrm{e}^{\beta\mu}) - N_p(s)\ln(1+\mathrm{e}^{\beta\mu'}) \tag{12}$$

$$= -(N - N_p(s))\ln(1+\mathrm{e}^{\beta\mu}) - N_p(s)\ln(1+\mathrm{e}^{\beta\mu'}) \tag{13}$$

$$= -N\ln(1+\mathrm{e}^{\beta\mu}) - N_p(s)\ln\left(\frac{1+\mathrm{e}^{\beta\mu'}}{1+\mathrm{e}^{\beta\mu}}\right) \tag{14}$$

where $N \equiv N_u(s) + N_p(s)$ is the sequence length. The first term is structure-independent and does not contribute to the populations in the experiment, so it can be safely ignored in our calculations. In practice, this formulation enables us to account for the probe influence on the secondary structure populations by adding $-\ln\left(\frac{1+\mathrm{e}^{\beta\mu'}}{1+\mathrm{e}^{\beta\mu}}\right)$ as a soft constraint in ViennaRNA for each paired base, allowing us to efficiently compute pairing probabilities within its computational framework. By substituting the expression for $\Delta F_{\text{[DMS]}}(s)$ into Equation 7, we obtain the final expression for the free energy of a secondary structure

$$F(s) = \underbrace{F_0(s)}_{\text{baseline}} + \underbrace{\sum_{i \in \text{paired}(s)} \lambda_i}_{\substack{\text{site-specific}\\ \text{corrections}}} \underbrace{- \mathrm{k_B} T N_p(s) \ln\left(\frac{1+\mathrm{e}^{\beta\mu'}}{1+\mathrm{e}^{\beta\mu}}\right)}_{\substack{\text{concentration}\\ \text{dependent perturbation}}} \tag{15}$$

Physical Binding Probability

Once we have outlined how to compute the populations of secondary structures in the presence of a chemical probe, we can derive the expected reactivity profile through the modeling of the chemical probing experiment, beginning from the interaction between the probe and the RNA. The probability of physical binding between the probe and RNA follows a Boltzmann distribution:

$$\mathbb{P}(k_i = 1|s_i = 0) = \frac{\mathrm{e}^{\beta\mu}}{1 + \mathrm{e}^{\beta\mu}} \tag{16}$$

$$\mathbb{P}(k_i = 1|s_i = 1) = \frac{\mathrm{e}^{\beta\mu'}}{1 + \mathrm{e}^{\beta\mu'}} \tag{17}$$

where $k_i \in \{0, 1\}$ denotes the physical binding state at position i (1 for probe-bound, 0 for unbound) and $s_i \in \{0, 1\}$ represents the pairing state (1 for paired, 0 for unpaired).

We make two key assumptions in this model: first, that the probe physical interaction is independent of nucleotide identity, and second, that binding events occur independently at each site, with no interactions between probes. Note that the latter may not hold at high probe concentrations, where cooperative effects and non-linear increases in reactivity can occur, as investigated by (22) and (56).

Probability of Chemical Modification

The probability of chemical modification at position i depends on both the physical binding event and the subsequent conversion from physical to chemical modification. We assume that when a probe physically interacts with nucleobase i, chemical modification occurs with a probability $p_{\mathsf{bind}}(n_i, s_i)$, which depends only on the nucleotide identity $n_i \in$ {A, U, G, C} and its pairing state s_i:

$$\begin{aligned}\mathbb{P}(c_i = 1|s_i, n_i) &= \mathbb{P}(c_i = 1|k_i = 1, s_i, n_i)\mathbb{P}(k_i = 1|s_i) && (18)\\ &\equiv p_{\mathsf{bind}}(n_i, s_i)\mathbb{P}(k_i = 1|s_i) && (19)\end{aligned}$$

where $c_i \in \{0, 1\}$ represents the modification state (1 for chemically modified, 0 for unmodified). While this is the most general formulation and holds for widely used chemical probes such as SHAPE, we note that DMS can react only with unpaired A and C and in a structure independent manner with G and U. We can thus simplify the model and embed the physics of the probe in the model by setting

$$\begin{aligned}p_{\mathsf{bind}}(\mathsf{A}, 1) &= 0\\ p_{\mathsf{bind}}(\mathsf{C}, 1) &= 0\\ p_{\mathsf{bind}}(\mathsf{G}, 0) &= p_{\mathsf{bind}}(\mathsf{G}, 1)\\ p_{\mathsf{bind}}(\mathsf{U}, 0) &= p_{\mathsf{bind}}(\mathsf{U}, 1)\end{aligned}$$

This leads to a total of four free parameters for the nucleotide-specific binding probabilities, one for each nucleotide type.

The overall probability of chemical modification at site i, denoted $\mathbb{P}(c_i)$, is obtained by averaging over the structural ensemble. Thanks to the assumption that binding and modification events are independent at each site, this average can be computed using the local pairing probabilities $\mathbb{P}(s_i)$, which are derived from the partition function without enumerating all possible secondary structures. The ensemble-averaged probability of chemical modification is thus given by:

$$\begin{aligned}\mathbb{P}(c_i) &= \langle \mathbb{P}(c_i|s, n_i)\rangle_s\\ &= \langle \mathbb{P}(c_i|s_i, n_i)\rangle_{s_i}\\ &= \sum_{s_i \in \{0,1\}} \mathbb{P}(s_i)\mathbb{P}(c_i|s_i, n_i) && (20)\end{aligned}$$

Expected Mutation Rate and Systematic Biases

In Mutation Profiling (MaP), chemical modifications induced by probes are detected as mutations in the cDNA during reverse transcription. To accurately model this process, we must establish the relationship between chemical modifications and the observed mutations while accounting for errors introduced during reverse transcription.

Although mutation probabilities could potentially depend on both sequence context and structural environment (24), we simplify our model by considering only the dependence on the chemical modification state c_i, treating other factors as systematic biases (which will be accounted for later in this section). We introduce two parameters $m(c_i = 1)$ and $m(c_i = 0)$ that govern respectively mutation probabilities for modified and unmodified nucleotides, according to the following definition:

$$\mathbb{P}(\text{mutation at position } i \mid c_i) \equiv 1 - \mathrm{e}^{-(m(c_i)+\epsilon_i)} \tag{21}$$

In this formulation, $m(c_i = 1)$ and $m(c_i = 0)$ can be interpreted as sequence-independent probabilities governing respectively false negative and false positive mutations and will be fitted as model parameters. In the following and in the main text we use the shorthand notation $m_1 \equiv m(c_i = 1)$ and $m_0 \equiv m(c_i = 0)$. The position-specific ϵ_i capture systematic biases common across all experimental replicates and probe concentrations for the same RNA sequence. These ϵ_i values are calibrated to ensure that the model-predicted reactivity profile at zero probe concentration aligns with the control experiment, effectively serving as site-specific offsets common to all experimental conditions.

To compute the model prediction of the experimental reactivity profile, we average over all possible modification states, weighted by their probabilities:

$$\begin{aligned}M_i &\equiv \langle M(c_i)\rangle_{c_i} && (22)\\ &= \sum_{j\in\{0,1\}} \mathbb{P}(c_i = j)M(c_i = j) && (23)\\ &= \sum_{j\in\{0,1\}} \mathbb{P}(c_i = j)(1 - \mathrm{e}^{-(m(j)+\epsilon_i)}) && (24)\\ &= 1 - \mathrm{e}^{-\epsilon_i} \sum_{j\in\{0,1\}} \mathbb{P}(c_i = j)\mathrm{e}^{-m_j} && (25)\\ &= 1 - \mathrm{e}^{-\epsilon_i}\left[(1 - \mathbb{P}(c_i = 1))\mathrm{e}^{-m_0} + \mathbb{P}(c_i = 1)\mathrm{e}^{-m_1}\right] && (26)\\ &= 1 - \mathrm{e}^{-\epsilon_i}\left[\mathrm{e}^{-m_0} + \mathbb{P}(c_i = 1)(\mathrm{e}^{-m_1} - \mathrm{e}^{-m_0})\right] && (27)\end{aligned}$$

This equation establishes a critical link between experimentally observed mutation rates and the underlying RNA structural

ensemble through the chemical modification probabilities $\mathbb{P}(c_i = 1)$, which themselves depend on the pairing state probabilities $\mathbb{P}(s_i)$ derived earlier.

Notably, the first term in the square brackets, containing m_0, produces a common background signal. When control experiments are available, this term can be incorporated into the systematic biases ϵ_i; otherwise, it must be fitted as a constant background term.

Model Parameters Summary

Before moving on to how to obtain the optimal model parameters from experimental data, we summarize here all the parameters of our model that require fitting:

- **Physical parameters**, which describe the physics of the chemical probing experiment. These are shared across all RNA sequences and probe concentrations.
  - μ_r: quantifies the energy of probe-RNA interaction at a reference probe concentration, from which we derive chemical potentials for all other concentrations.
  - $\Delta\mu_{\text{pairing}}$: represents the energetic penalty for probe interaction with paired nucleobases.
  - $p_{\text{bind}}(n_i, s_i)$: is the probability of chemical modification given nucleotide identity n_i and pairing state s_i. These are four for DMS, one for each nucleotide type.
  - m_1 and m_0: are parameters governing respectively false negative and false positive mutation probabilities during the cDNA transcription. If control experiments are available, m_0 gets absorbed into the systematic biases ϵ_i and does not need to be fitted.
- **Soft constraints**, which are sequence-specific but consistent across all experimental conditions for a given RNA:
  - λ_i: are site-specific soft constraints modifying the pairing energy of the baseline thermodynamic model.

Worth mentioning in this summary–although not fitted–are the ϵ_i, which are calibrated from the control experiment and incorporate site-specific systematic errors shared across replicates and concentrations, and effectively serve as offsets to the predicted mutation profiles. They enable the decoupling of experimental biases from the thermodynamic and concentration-dependent effects, allowing the model to focus on physically relevant signal.

Computation of base-pairing probabilities

All base-pairing probabilities (BPPs) reported in this work were computed with ViennaRNA v2.7.0 (20) through its Python interface, using the default Turner 2004 nearest-neighbour parameters (37) unless otherwise stated; results with the Andronescu 2007 parameters (38), loaded via `RNA.params_load_RNA_Andronescu2007()`, are reported alongside where indicated. For each sequence the folding temperature is set with (`RNA.cvar.temperature`) and we build a `fold_compound`; any per-nucleotide energy correction is added to this `fold_compound` first, and only then do we evaluate the partition function (`fc.pf()`) and read the equilibrium BPP matrix (`fc.bpp()`). The three models compared in Figure 2 share this identical pipeline and differ only in the correction applied before `fc.pf()`:

- **ViennaRNA baseline** (reactivity-agnostic): no correction; the unmodified thermodynamic ensemble.
- **Deigan pseudo-free-energy** (14): after subtraction of the control and clipping of negatives to zero, reactivities are normalised by the standard box-plot procedure (values above the $Q_3 + 1.5\,\mathrm{IQR}$ outlier fence are discarded and the profile is divided by the mean of the top 10% of the remaining values), computed over the probed adenine and cytosine positions only, then converted to a per-position pseudo-free-energy through ViennaRNA's dedicated `fc.sc_add_SHAPE_deigan(reactivities, m, b)`, applied to adenines and cytosines only. Rather than adopting the literature defaults $(m, b) = (1.8, -0.6)$, the slope and intercept were refit to our dataset, as detailed in the SI Appendix (Refitting the Deigan slope and intercept).
- **MERGE-RNA**: during the fit we apply, on top of the baseline, both the inferred site-specific soft constraints λ_i and the concentration-dependent penalty on paired bases $\Delta F_{\text{[DMS]}}$ (Equation 3), which acts as a uniform free-energy penalty applied to every paired nucleotide. Both enter through the same ViennaRNA soft-constraint interface: a per-nucleotide energy can be added equivalently either to a base's unpaired state (`sc_add_up`) or to all of its base pairs (`sc_add_bp`), the two yielding the same normalized ensemble, and we choose between them for numerical stability (for the λ_i, according to their sign). The extrapolated BPPs at zero probe concentration are then obtained by setting $\Delta F_{\text{[DMS]}} = 0$. To optimize the model parameters, derivatives of the base-pairing probabilities with respect to the optimized parameters are computed from conditional pairing probabilities obtained by imposing hard constraints that force individual nucleotides to remain unpaired (`hc_add_up`).

In all cases the corrections enter the ViennaRNA recursions directly, so the partition function is recomputed with the modified energies and the BPPs reflect the full, exactly re-weighted Boltzmann ensemble. We do not enumerate or re-score a finite set of suboptimal structures a posteriori. Should individual representative structures be of interest, they can be obtained downstream from the same constrained ensemble (e.g. by stochastic backtracking or `RNA.fold_compound.subopt`); while this can be useful for downstream analyses, it plays no role in the fit.

Model Training

Loss function

To fit the model parameters to experimental data, we define a loss function that quantifies the discrepancy between the expected reactivity profiles predicted by our model (as derived in the previous section) and the observed mutation rates from chemical probing experiments.

The binomial nature of the mutation counts, which arise from a series of independent Bernoulli trials (each read can either show a mutation or not), motivates the choice of a binomial likelihood for our loss function. Within this framework, the probability of observing $\mathcal{M}_i$ mutations out of a coverage of n_i

reads under predicted mutation rate M_i is given by:

$$\mathbb{P}(\mathcal{M}_i) = \text{Binomial}(\mathcal{M}_i, n_i, M_i) \tag{28}$$

$$= \binom{n_i}{\mathcal{M}_i} M_i^{\mathcal{M}_i}(1-M_i)^{n_i-\mathcal{M}_i} \tag{29}$$

where i runs over all the data points, i.e. sequences, positions, concentrations and replicates. We thus define the loss function as the negative log-likelihood of observing the experimental mutation counts $\mathcal{M}_i^{\text{exp}}$ given the model.

$$\mathcal{L} = \sum_i -\ln \mathbb{P}(\mathcal{M}_i^{\text{exp}} = \mathcal{M}_i) \tag{30}$$

$$= \sum_i -\ln\left[\binom{n_i}{\mathcal{M}_i^{\text{exp}}} M_i^{\mathcal{M}_i^{\text{exp}}}(1-M_i)^{n_i-\mathcal{M}_i^{\text{exp}}}\right] \tag{31}$$

Fit of parameters

The model fitting process involves optimizing a set of parameters to best reproduce experimental data. We implemented the optimization procedure in a custom Python script utilizing the `scipy.optimize.minimize` function (26) with the L-BFGS-B algorithm (25). This limited-memory quasi-Newton method is particularly well-suited for high-dimensional optimization problems, as it efficiently approximates the inverse Hessian matrix while maintaining reasonable memory requirements. The algorithm's ability to handle bound constraints is especially valuable for ensuring physically meaningful parameter values throughout the optimization process. Specifically, we constrained the parameters to physically plausible ranges: the chemical potential at reference concentration, μ_r, was bounded between -5 and 5 kcal/mol; the energetic penalty for pairing, $\Delta\mu_{\text{pairing}}$, was constrained to be non-negative, with an upper bound of 10 kcal/mol; the chemical modification probabilities, p_{bind}, were constrained to the range $[0, 1]$, as is required for probabilities; the false positive mutation parameter, m_0, was kept small within $[0, 0.1]$; and the false negative parameter, m_1, was bounded within $[0.1, 10]$.

The optimization is performed in a two-stage process to minimize the loss function. In the first stage, we fit the physical parameters that are common to all experiments, keeping the sequence-specific soft constraints (λ_i) fixed. This global optimization starts from random initializations and runs until convergence. In the second stage, the optimized physical parameters are held constant while we fit the λ_i soft constraints independently for each RNA sequence. This hierarchical strategy allows us to first establish a robust physical model and then refine the secondary structure populations with sequence-specific adjustments. During the optimization, the λ_i parameters are constrained to the range $[-1, 1]$ kcal/mol to ensure proper regularization and avoid overfitting.

Experimental Procedures

In vitro transcription (IVT)

A single-stranded full-length DNA oligonucleotide (`5'-ATGGTCTGCTGGAGGTTGGAATAGTTGTGAGTTGAAGTGGGGATTTTTTAATCCCCACTTCAACTCACAACTATTCCAATTTTTTGGAATAGTTGTGAGTTGAAGTGGGGATTGGCTGTGGCATAATG-3'`) was chemically synthesized (Integrated DNA Technologies). This full-length sequence was amplified by PCR using primers carrying a T7 promoter. The primers used were Full-T7-RV (`5'-TAATACGACTCACTATAGGGCATTATGCCACAGCCAATCCCCACTTC-3'`) and Full-FW (`5'-ATGGTCTGCTGGAGGTTGGAATAGTTGTGAG-3'`).

PCR was performed in 50 µL reactions containing the following final concentrations: GXL reaction buffer (1×, from a 5× stock; Takara Bio), each dNTP (0.2 mM), each primer (0.25 µM; Full-T7-RV and Full-FW), single-stranded full-length DNA template (1 nM), and PrimeSTAR GXL DNA Polymerase (0.025 U/µL; Takara Bio). Thermal cycling conditions were as follows: initial denaturation at 98 °C for 2 min; 25 cycles of 98 °C for 20 s, 55 °C for 20 s, and 68 °C for 30 s; followed by a final extension at 68 °C for 5 min. PCR products were analyzed on a 2% agarose gel to verify the expected size, before purification using the NucleoSpin Gel and PCR Clean-up kit with NTC buffer (Macherey–Nagel), according to the manufacturer's instructions. The purified DNA was used as a template for T7 RNA polymerase — mediated in vitro transcription (IVT). IVT was performed in 50 µL reactions containing the following final concentrations: T7 RNA Polymerase Reaction Buffer (1×, from a 10× stock; New England Biolabs), ATP, CTP, GTP and UTP (each 5 mM; Ribonucleotide Solution Mix, New England Biolabs), RNase Inhibitor, Murine (1 U/µL; New England Biolabs), T7 RNA Polymerase (2.5 U/µL; New England Biolabs), DNA template (25 nM), and inorganic pyrophosphatase (0.0005 U/µL; Thermo Scientific), in nuclease-free water. Reactions were incubated at 37 °C for 2 h. Positive IVT control reactions were set up identically, except that a previously validated DNA template was used. After IVT, residual DNA template was removed by DNase digestion. DNase digestion was carried out at 37 °C for 30 min in reactions containing the following final concentrations: DNase TURBO (0.1 U/µL) and DNase TURBO buffer (1×; Invitrogen). IVT products with and without DNase treatment were analyzed on a 2% agarose gel to assess the efficiency of DNA removal and the quality of the RNA transcripts.

RNA refolding

DNase-treated IVT products were column-purified and used for RNA refolding. For the initial refolding step, RNA was diluted in ultrapure water to an RNA concentration of 26 nM, and refolding buffer was added to reach the following final concentrations: EDTA (0.5 mM), HEPES (200 mM, pH 7.5), NaCl (300 mM). The mixture was heated at 95 °C for 1.5 min to denature RNA secondary structures and then immediately placed on ice. While on ice, $MgCl_2$ was added to give a final concentration of 5 mM and an RNA concentration of 23.5 nM. Samples were then incubated at 37 °C for 30 min to allow RNA refolding. Refolded RNA was stored at -80 °C until further use.

DMS probing

For dimethyl sulfate (DMS) probing, reactions were prepared with refolded RNA at a final concentration of 23.5 nM. DMS (Sigma–Aldrich) stock solutions were prepared in 100% ethanol such that, upon addition to the RNA samples, the final DMS concentrations were 20, 40, 60, 80, or 100 mM. β-mercaptoethanol (Sigma–Aldrich) was diluted in PBS such that, upon addition to the reaction, its final concentration was 1000 mM. For each DMS concentration, one negative control (receiving an equal volume of 100% ethanol instead of DMS)

and two technical replicates with DMS were prepared. Refolded RNA samples were kept on ice, mixed thoroughly with the appropriate DMS working solution or with 100% ethanol for negative controls, and immediately transferred to an incubator pre-equilibrated to 37 °C for 7 min. Reactions were then placed back on ice, and β-mercaptoethanol working solution was added to each reaction and mixed thoroughly to reach its final concentration of 1000 mM and to quench DMS modification.

Reverse transcription

DMS-treated RNA was column-purified and subjected to reverse transcription using MarathonRT (28; 16; 29). Primer–RNA annealing reactions were prepared on ice with the following final concentrations: each dNTP (0.5 mM) and Full-FW primer (0.25 µM). Primer–RNA mixtures were incubated at 65 °C for 5 min and then rapidly cooled on ice. Reverse transcription was then performed in 40 µL reactions containing the primer–RNA annealing mixture and the following final concentrations: MarathonRT reaction buffer (1×; final composition 50 mM Tris–HCl, pH 8.3; 200 mM KCl; 20% (v/v) glycerol, prepared from a 3× stock containing 150 mM Tris–HCl, pH 8.3; 600 mM KCl; 60% (v/v) glycerol), $MnCl_2$ (1 mM), MarathonRT (1 U/µL; in-house purified, Addgene plasmid no. 109029 (27); http://n2t.net/addgene:109029; RRID: Addgene_109029; stored in 50 mM Tris–HCl, pH 8.3; 200 mM KCl; 20% (v/v) glycerol), RNase Inhibitor, Murine (1 U/µL; New England Biolabs), and DTT (5 mM). No–reverse transcriptase (no–RT) controls were prepared in parallel with the same components and final concentrations, except that MarathonRT was omitted.

PCR amplification

cDNA products were column-purified and subsequently amplified by PCR. PCR amplification was performed in 50 µL reactions containing the following final concentrations: GXL reaction buffer (1×, from a 5× stock; Takara Bio), each dNTP (0.2 mM), each primer (0.25 µM; Full-T7-RV and Full-FW), and PrimeSTAR GXL DNA Polymerase (0.025 U/µL; Takara Bio). An appropriate amount of cDNA was used as template. Thermal cycling conditions were: 98 °C for 2 min; 25 cycles of 98 °C for 20 s, 55 °C for 20 s, and 68 °C for 30 s; followed by a final extension at 68 °C for 5 min. PCR amplicons were analyzed on a 2% agarose gel and stained with ethidium bromide (EtBr) to assess amplification quality.

Nanopore sequencing

PCR amplicons were column-purified, and DNA concentrations were determined using the Qubit dsDNA HS Assay Kit (Thermo Fisher Scientific). After normalizing the molar concentration of each sample, a total of 1,600 fmol DNA was used in library preparation, with 5 µL taken from each sample. For each sample, dA-tailing and 5′ phosphorylation were performed in 6 µL reactions containing 5 µL DNA, 0.7 µL NEBNext End-Repair Buffer (1× final concentration; New England Biolabs), and 0.3 µL NEBNext End-Repair Enzyme Mix (New England Biolabs). Reactions were incubated at 20 °C for 10 min, followed by 65 °C for 10 min. Barcoding ligation was performed using the SQK–NBD114–96 kit (Oxford Nanopore Technologies). For each sample, barcoding ligation was carried out in 5 µL reactions containing 1.5 µL end-repaired DNA, 1 µL barcode, and 2.5 µL NEB Blunt/TA Ligase Master Mix (New England Biolabs, added as supplied). Reactions were incubated at room temperature for 20 min, after which ligation was quenched by adding 1 µL EDTA (SQK–NBD114–96). Subsequently, 5 µL of each barcoded sample was pooled, and the resulting pool was purified with 1× AMPure XP beads (SQK–NBD114–96) and washed twice with Short Fragment Buffer (SFB; SQK–NBD114–96). The pooled barcoded DNA was eluted in 35 µL nuclease-free water. Adapter and motor protein ligation was performed in 56 µL reactions containing 35 µL pooled barcoded DNA, 11 µL NEBNext Quick Ligation Reaction Buffer (New England Biolabs, B6058S; 1× final concentration), 5 µL Native Adapter (NA; SQK–NBD114–96, Oxford Nanopore Technologies), and 5 µL high-concentration NEB T4 DNA Ligase (New England Biolabs, T2020M). Reactions were incubated at room temperature for 20 min. The library was then purified with 0.6× AMPure XP beads (SQK–NBD114–96) and washed twice with SFB, taking care to avoid drying of the beads during washing and prior to elution. The final library was loaded onto an R10.4.1 flow cell (FLO–PRO114M; Oxford Nanopore Technologies) and sequenced on a PromethION 2 Solo instrument (Oxford Nanopore Technologies) controlled by MinKNOW software (version 25.09.16). Basecalling was performed with Dorado v1.1.1 (Oxford Nanopore Technologies) in super-accuracy ("sup") mode using the `dna_r10.4.1_e8.2_400bps_sup@v5.2.0` model.

Mapping and counting of mutations

Sequencing reads were processed with an RNAFramework-based (30) workflow using the functions `rf-map` and `rf-count`, following a similar pipeline to (18) with minor adaptations for our constructs. For each library, we generated a Bowtie2 (31) index from the reference sequence using `bowtie2-build` and produced the companion FASTA index with `samtools faidx` (32). Reads were aligned to the indexed reference with `rf-map -cq5 20 -cqo -mp '--very-sensitive-local' -b2 -bi ../{ref_fasta}_index`, where `{ref_fasta}_index` are the files produced by `bowtie2-build`. The resulting BAM files were coordinate sorted and indexed (`samtools sort/index`) before quantification.

Mutation profiles were obtained with `rf-count` (30) using the options recommended by Ref. (18) (`-m -ds 75 -na -ni -md 3`).These parameters report mutational load rather than RT stops (`-m`), discard reads shorter than 75 nt (`-ds 75`), ignore ambiguous deletions (`-na`) and indels (`-ni`), and cap deletions at three nucleotides (`-md 3`). The full command sequence executed for each FASTQ file is summarized below:

```
# build indices of reference
bowtie2-build --quiet {ref_fasta} {ref_fasta}_index
samtools faidx {ref_fasta}
# alignment (see above)
rf-map \
    -cq5 20 \
    -cqo \
    -mp '--very-sensitive-local' \
    -b2 \
    -bi {ref_fasta}_index \
    -o rf_map_{output_suffix} \
    {fastq_file}
```

```
# sorting and indexing the BAM file
samtools sort {bam_file} -o {sorted_bam_file}
samtools index {sorted_bam_file}
# count mutations to obtain mutation profile and mutation ma
rf-count \
    -f {ref_fasta} \
    -m \
    -ds 75 \
    -na \
    -ni \
    -md 3 \
    {sorted_bam_file}
```

For the reference structured RNAs (HCV IRES, bacterial RNase P, *V. cholerae* glycine riboswitch, *Tetrahymena* ribozyme, HC16), we instead adopted the filtering scheme described by the original work (16).

```
rf-count -r -p {num_threads} \
    -mf {primer_mask} \
    -f {reference_fasta} \
    --only-mut 'G>Y;A>B;C>D;T>V' \
    -m \
    -nd \
    -ni \
    -q {minq} \
    -eq 10
```

All downstream structural analyses reported in the main text rely on the coverage and mutation rates generated by this standardized workflow.

Importance of conformational heterogeneity in structured RNAs

We found it instructive to illustrate, with a simple controlled experiment, why ensemble heterogeneity is needed to explain chemical-probing data and where the compromise between structural specificity and conformational diversity lies. Using HCV IRES as a representative example, we applied a uniform soft constraint instead of inferring it position-by-position (Fig. S1, analogous results for other systems in Figs. S3,S4,S5): for a given scalar magnitude (x-axis), every nucleotide annotated as paired in the chosen reference structure (experimental or minimum-free-energy) was assigned that value, all other positions were set to zero, and the physical parameters were kept fixed. Increasing this magnitude progressively biases the ensemble toward the targeted structure, smoothly interpolating between the original thermodynamic ensemble and a single constrained structure.

By interpolating between the original ensemble and its single most-likely structure (minimum-free-energy), we assess the impact of reducing ensemble heterogeneity on the agreement with experiment (light green line in Fig. S1). In fact, favoring a single structure has the effect of progressively suppressing the population of alternative conformations that are likely present in solution and contribute to the observed mutation profiles. Interestingly, the loss function monotonically increases when the constraint is applied, degrading the agreement with experiment. On the other hand, incorporating knowledge from the reference experimental structure (red line in Fig. S1) initially improves

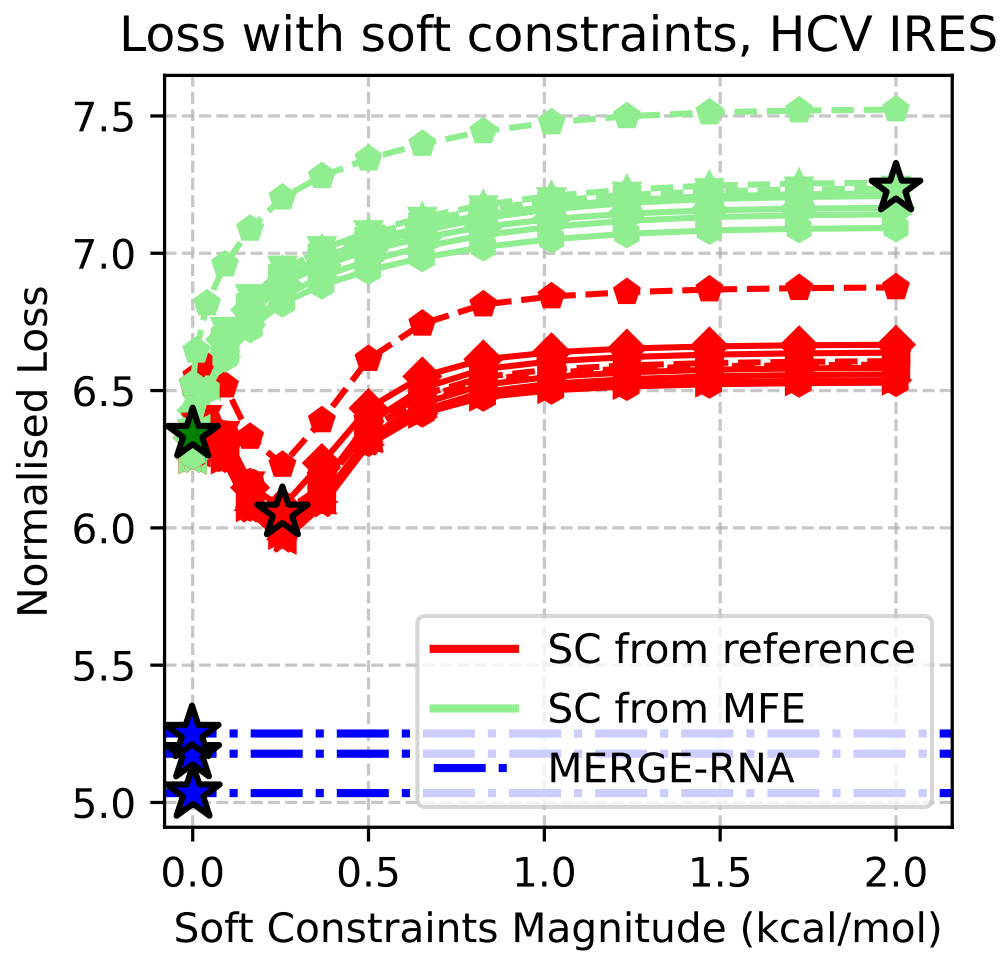

Figure S1 Effect of a uniform soft constraint on the agreement with chemical-probing data for HCV IRES. For each scalar magnitude on the x-axis, every nucleotide annotated as paired in the chosen reference structure is assigned that value as its soft constraint, all other positions are set to zero, and the physical parameters are kept fixed; this smoothly interpolates the ensemble between the original thermodynamic prediction and a single constrained structure. The y-axis reports the per-datapoint loss (Equation 31). Red line: constraints derived from the experimental reference structure; light-green line: constraints derived from the minimum-free-energy (MFE) structure predicted by the thermodynamic model. Each curve corresponds to an independent fit in which the physical parameters were trained on a different triplet of RNA systems (cross-validation sets of Fig. 2a). The horizontal blue line indicates the loss reached by MERGE-RNA when the sequence-specific λ_i are inferred position-by-position. The non-monotonic behavior of the red curve, together with the lower loss attained by the blue line, illustrates that explaining the observed mutation profiles requires retaining ensemble heterogeneity rather than collapsing onto any single structure, and that this is most effectively achieved by inferring per-nucleotide soft constraints.

the model's agreement with experimental data. However, even in this case, excessive constraint enforcement eventually degrades performance. This pattern indicates the existence of a "sweet spot" where the model partially incorporates reference structural information while preserving the conformational diversity necessary to explain the experimental measurements. Too strong a constraint toward any single structure—even the experimental reference—reduces the model's ability to account for the heterogeneous structural ensemble that is captured in the experimental data. Overall, this analysis shows that a proper balance of information from the reference single experimental structure and ensemble diversity improves the capability of the model to reproduce chemical probing experiments.

Refitting the Deigan slope and intercept

The Deigan pseudo-free-energy method (14) enters our benchmark through exactly the same ViennaRNA folding pipeline as MERGE-RNA, described above under "*Computation of base-pairing probabilities*": a reactivity r_i is converted into the per-nucleotide restraint $\Delta G_i = m\,\ln(1 + r_i) + b$ and added to the folding free energy with `sc_add_SHAPE_deigan`, applied

to adenines and cytosines only. Only two ingredients are specific to this baseline: the reactivities passed to the restraint and the coefficients (m, b).

Input reactivities.

We start from the same mutation-rate data used by MERGE-RNA and subtract the untreated control, clip negative values to zero. The resulting reactivities are normalised following the standard box-plot normalisation scheme: values above the $Q_3 + 1.5\,\mathrm{IQR}$ box-plot outlier fence are discarded and the profile is divided by the mean of the top 10% of the remaining values, before passing it to `sc_add_SHAPE_deigan`. Because DMS probes only adenine and cytosine, this normalisation factor is computed over the A/C positions only. All downstream comparisons use the same per-position masking as the corresponding MERGE-RNA fits, so that the two methods see identical data.

Selection of the coefficients (m, b).

The slope and intercept are usually taken from the literature, but their optimum depends on the probe, the experimental conditions and the thermodynamic model, and is commonly re-optimized on the dataset at hand (18). We borrow the grid-search selection of RNAFramework's `rf-jackknife` (30): we scan $m \in [0, 5]$ and $b \in [-3, 0]$ with step 0.2 (416 combinations) and, for each pair, fold every system with the Deigan restraint and score the predicted minimum-free-energy structure against the reference by the relaxed Fowlkes–Mallows index ($\mathrm{FMI} = \sqrt{\mathrm{PPV} \cdot \mathrm{sensitivity}}$, ± 1-nt tolerance, pseudoknots and lonely pairs removed), as previously done in (18). The (m, b) pair with the highest geometric mean of the FMI over all systems is selected as the optimum. For the structured RNAs of Fig. 2 the reference structures are themselves the evaluation target, so choosing (m, b) on them would be circular; we therefore select the coefficients by leave-one-system-out at each DMS concentration: for each held-out system, (m, b) is optimized on the other three at the same concentration and applied to the held-out one, obtaining one coefficient pair per concentration and per held-out system. The joint per-concentration optimum is given in Table S1; Table S2 reports instead, for every system and concentration, the per-held-out-system coefficients used for the Fig. 2 bars and the two oracle performance ceilings (Frobenius and probing R^2, defined below). For the other RNAs analysed in this work no single reference structure is available, so the protocol above is not applicable. Since those experiments were all probed at $[\mathrm{DMS}] \geq 57$ mM, we reuse the 57 mM grid optimum over the four PDB-referenced systems (without any leave-out) as a fixed, transferable coefficient, $(m, b) = (2.4, -0.4)$ under Turner and $(2.4, -1.0)$ under Andronescu.

Performance ceiling.

In the main text the solid Deigan bars use the coefficients selected above (held-out for the structured RNAs, transferable for the others). The dashed caps instead report the *ceiling* of the Deigan functional form: on the same (m, b) grid we score each pair directly with the metric of interest: the Frobenius distance in Fig. 2b and the probing R^2 in Fig. 2d, in place of the FMI used for selection and keep its optimum, which is by construction the best value any choice of (m, b) can attain for that metric, and therefore an oracle upper bound on what this two-parameter form can achieve.

Table S1. Joint Deigan optimum: slope and intercept (m, b) that maximize the geometric-mean FMI over all four PDB-referenced systems at each DMS concentration (per-concentration, no leave-out), under the Turner 2004 and Andronescu 2007 baselines. The per-system, held-out and oracle-ceiling coefficients are reported in Table S2; the literature default is $(1.8, -0.6)$.

$[\mathrm{DMS}]$ (mM)	Turner (m, b)	Andronescu (m, b)
8	$(0.0, \ 0.0)$	$(1.4, -0.2)$
17	$(2.0, \ 0.0)$	$(1.0, \ 0.0)$
34	$(1.8, \ 0.0)$	$(4.0, -0.6)$
57	$(2.4, -0.4)$	$(2.4, -1.0)$

Combination of experiments performed in different settings: cspA 5' UTR

We here test the capability of MERGE-RNA to operate in situations where experiments performed in different settings are combined, namely in capturing complex structural rearrangements by considering two DMS mutational profiles for *cspA* 5′ UTR recorded at 10 and 37°C (42). This sequence acts as a thermoswitch (41), with different structures at the two reported temperatures. The two experiments have been recorded at different probing times (2 hours vs 10 minutes). Our approach has been designed to analyze homogeneous experiments and does not model the dependence of the observed mutation rate on the probing time. We hence normalize the dataset at 10°C to the same average mutation rate observed at 37°C. In addition, to avoid confusing the effect of the mutational profiles with the temperature-dependence of the thermodynamic model, we use the thermodynamic model set on the same intermediate temperature for every ensemble (23.5°C). Finally, we note that these experimental datasets lack a control mutation profile obtained in absence of DMS, making the separation of the physical signal from the systematic bias more difficult for our model. Given the mentioned limitations, we decided to perform a set of 6 independent fits, starting from different random initializations of the physical parameters. We report in Fig. S2 the ensemble predicted by the model that achieved the lowest loss, while additional fits from independent random initializations are shown in Figs S24–S28.

In Fig. S2(a,b), the structural ensembles predicted by MERGE-RNA largely recapitulate the individual structures reported by Zhang et al. (42). At 10°C and 37°C, our ensembles closely match their predictions in positions 1–120 and 70–170, respectively, but exhibit notable differences in other regions, particularly in segments where alternative helices compete. Unlike Ref. (42), which reports a single minimum-free-energy structure, MERGE-RNA predicts a thermodynamic ensemble that accounts for multiple competitive helices and their relative populations. Overall, this analysis demonstrates that our model can capture complex structural rearrangements in biological systems. For comparison, the corresponding predictions obtained with the optimized Deigan pseudo-free-energy approach are shown in Fig. S29.

Table S2. Deigan slope and intercept (m, b) for every PDB-referenced system and DMS concentration, under the Turner 2004 and Andronescu 2007 baselines, obtained on the $m \in [0, 5]$, $b \in [-3, 0]$ grid (step 0.2). *Held-out*: leave-one-system-out optimum used for the Fig. 2 bars, optimized on the other three systems at the same concentration. *Ceiling*$_F$ and *Ceiling*$_{R^2}$: oracle optima that instead minimize the Frobenius distance (Fig. 2b) or maximize the probing R^2 (Fig. 2d) directly on the same grid, i.e. the best value the two-parameter form can attain for that metric. The joint per-concentration optimum is reported separately in Table S1; the literature default is $(m, b) = (1.8, -0.6)$.

System	[DMS] (mM)	Held-out (m, b)	Ceiling$_F$ (m, b)	Ceiling$_{R^2}$ (m, b)
Turner 2004				
hc16 ligase	8	$(0.0, 0.0)$	$(0.6, -0.4)$	$(0.6, -0.4)$
	17	$(2.0, 0.0)$	$(1.2, 0.0)$	$(1.8, -0.2)$
	34	$(1.8, 0.0)$	$(1.6, -0.2)$	$(3.4, -0.6)$
	57	$(2.2, -0.4)$	$(1.4, -0.6)$	$(3.6, -1.2)$
Tetrahymena ribozyme	8	$(1.4, 0.0)$	$(0.4, 0.0)$	$(1.6, -0.2)$
	17	$(2.6, 0.0)$	$(0.2, 0.0)$	$(1.2, -0.2)$
	34	$(4.4, -0.2)$	$(0.2, 0.0)$	$(1.8, -0.6)$
	57	$(2.4, -0.4)$	$(0.2, 0.0)$	$(3.0, -0.8)$
HCV IRES	8	$(0.6, -0.8)$	$(0.0, -0.2)$	$(0.2, 0.0)$
	17	$(2.0, 0.0)$	$(4.0, 0.0)$	$(2.8, 0.0)$
	34	$(4.4, -1.2)$	$(5.0, 0.0)$	$(4.6, -0.8)$
	57	$(3.4, -1.4)$	$(2.2, 0.0)$	$(2.0, 0.0)$
V. cholerae gly. riboswitch	8	$(0.0, 0.0)$	$(2.0, -0.4)$	$(2.2, -0.6)$
	17	$(2.6, 0.0)$	$(2.4, -0.2)$	$(3.6, -0.8)$
	34	$(1.8, 0.0)$	$(2.0, 0.0)$	$(2.6, -3.0)$
	57	$(2.8, -0.2)$	$(2.2, -0.4)$	$(2.0, -2.2)$
Andronescu 2007				
hc16 ligase	8	$(1.4, -0.2)$	$(1.8, -0.2)$	$(0.8, -0.8)$
	17	$(0.8, 0.0)$	$(2.0, -0.2)$	$(3.8, -0.4)$
	34	$(1.4, 0.0)$	$(3.6, -0.6)$	$(4.0, -0.6)$
	57	$(1.4, -0.6)$	$(2.8, -1.0)$	$(5.0, -1.6)$
Tetrahymena ribozyme	8	$(1.4, -0.2)$	$(0.0, 0.0)$	$(1.6, -0.4)$
	17	$(1.4, 0.0)$	$(0.0, 0.0)$	$(0.0, -1.0)$
	34	$(4.4, -0.6)$	$(0.0, 0.0)$	$(0.0, -1.0)$
	57	$(2.4, -1.0)$	$(0.0, 0.0)$	$(4.8, -1.2)$
HCV IRES	8	$(1.6, -0.4)$	$(0.6, 0.0)$	$(0.6, 0.0)$
	17	$(0.6, 0.0)$	$(0.6, 0.0)$	$(0.6, 0.0)$
	34	$(2.4, -0.6)$	$(3.2, -0.2)$	$(0.8, 0.0)$
	57	$(3.6, -1.2)$	$(1.0, 0.0)$	$(0.8, 0.0)$
V. cholerae gly. riboswitch	8	$(0.2, -0.2)$	$(1.0, 0.0)$	$(0.0, 0.0)$
	17	$(1.0, 0.0)$	$(1.8, -0.2)$	$(1.2, -0.8)$
	34	$(4.0, -0.6)$	$(1.6, 0.0)$	$(1.6, -1.0)$
	57	$(2.0, -1.0)$	$(2.0, -0.6)$	$(0.4, -0.2)$

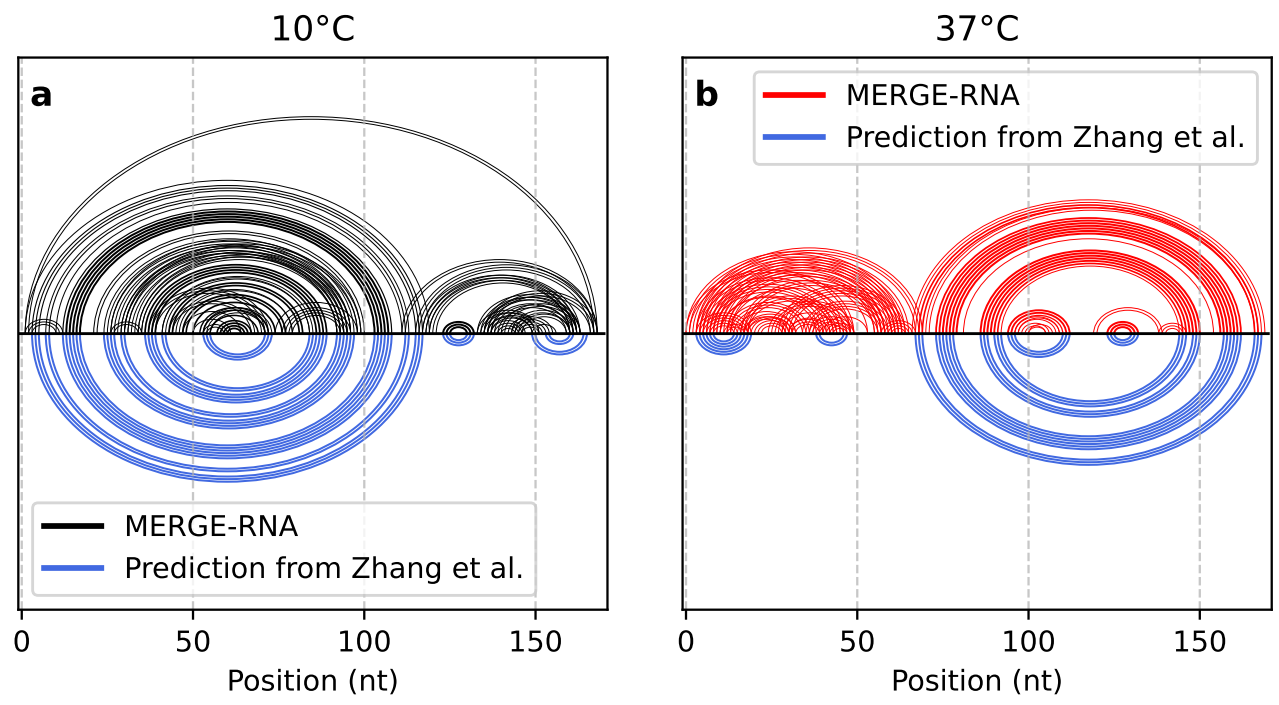

Figure S2 Ensemble predictions for *cspA* 5′ UTR at two different temperatures. **(a,b)** Arc plots corresponding to the DMS data collected at 10 and 37°C, respectively. Predictions from MERGE-RNA (red and black) are compared with predictions from Ref. (42) (blue).

Supplementary Figures and Tables

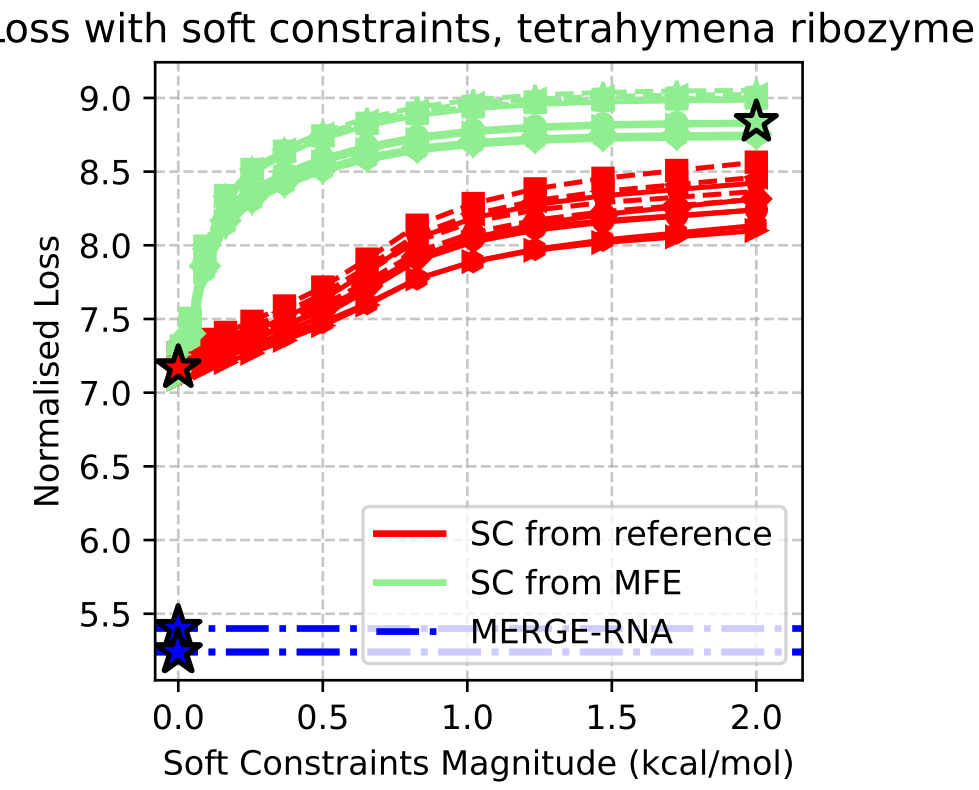

Figure S3 Same analysis as in Fig. S1, for the *Tetrahymena* ribozyme.

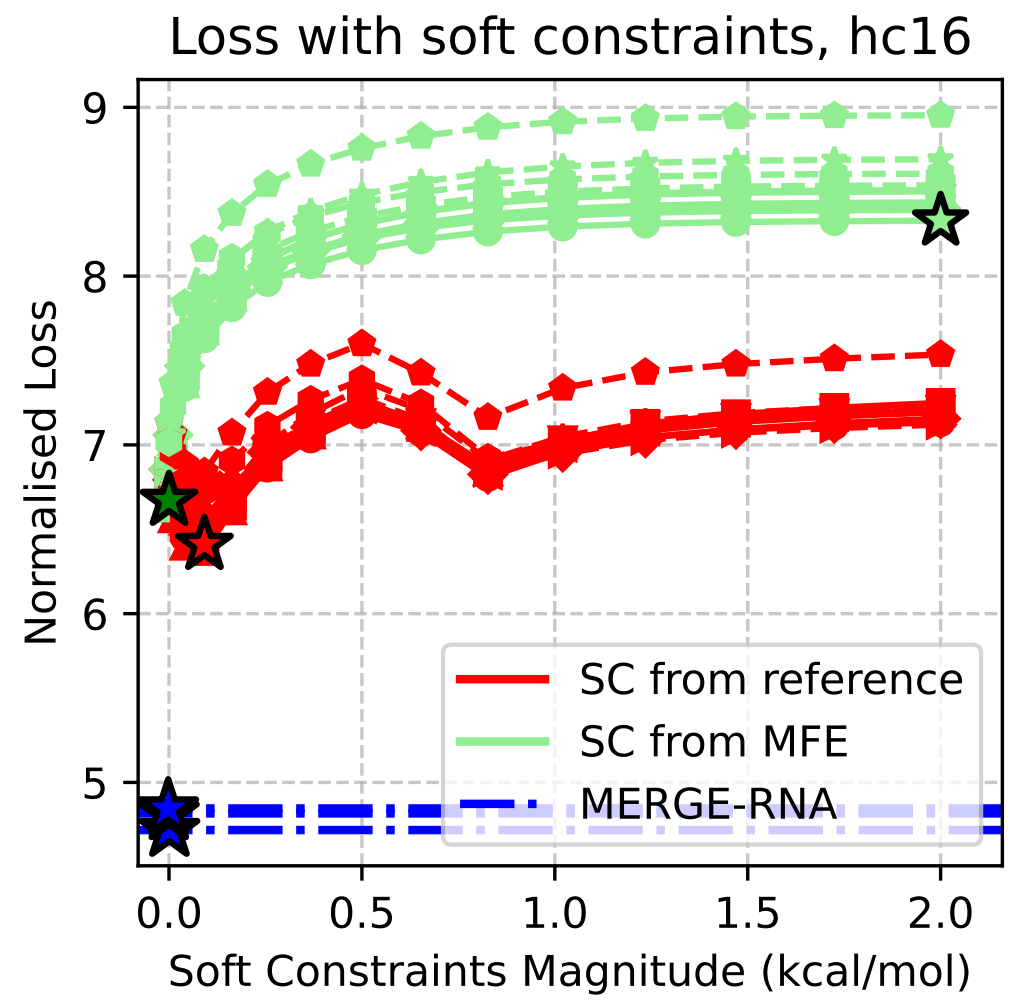

Figure S5 Same analysis as in Fig. S1, for the hc16 ligase.

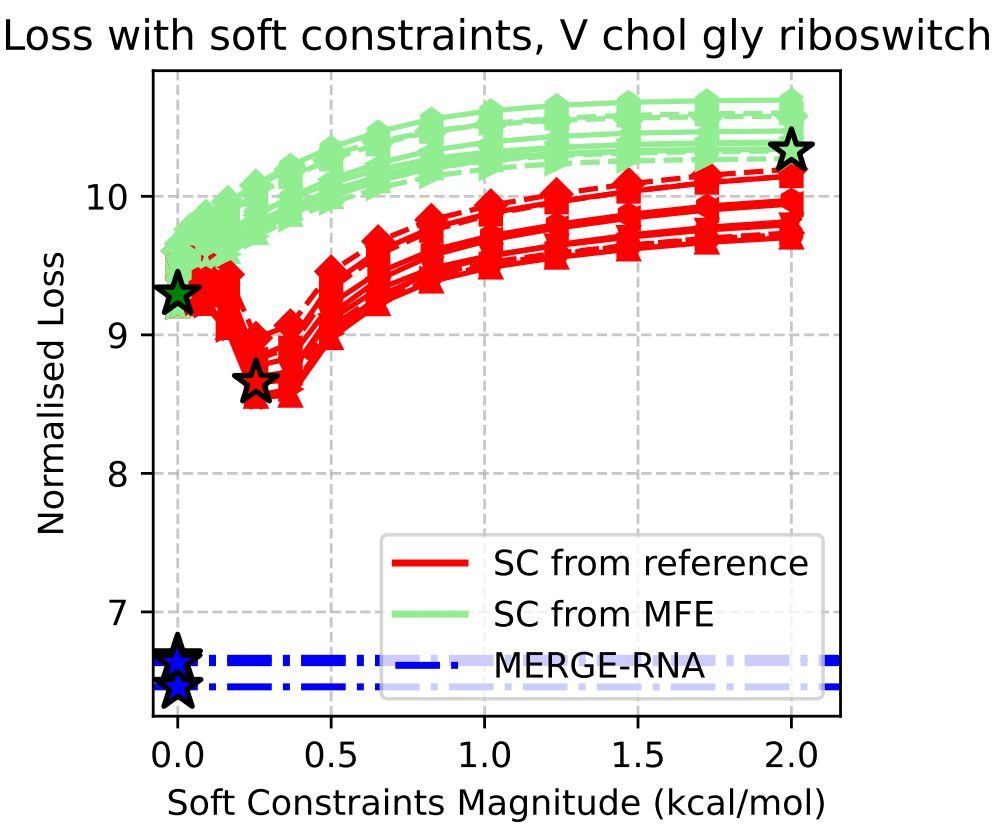

Figure S4 Same analysis as in Fig. S1, for the *V. cholerae* glycine riboswitch.

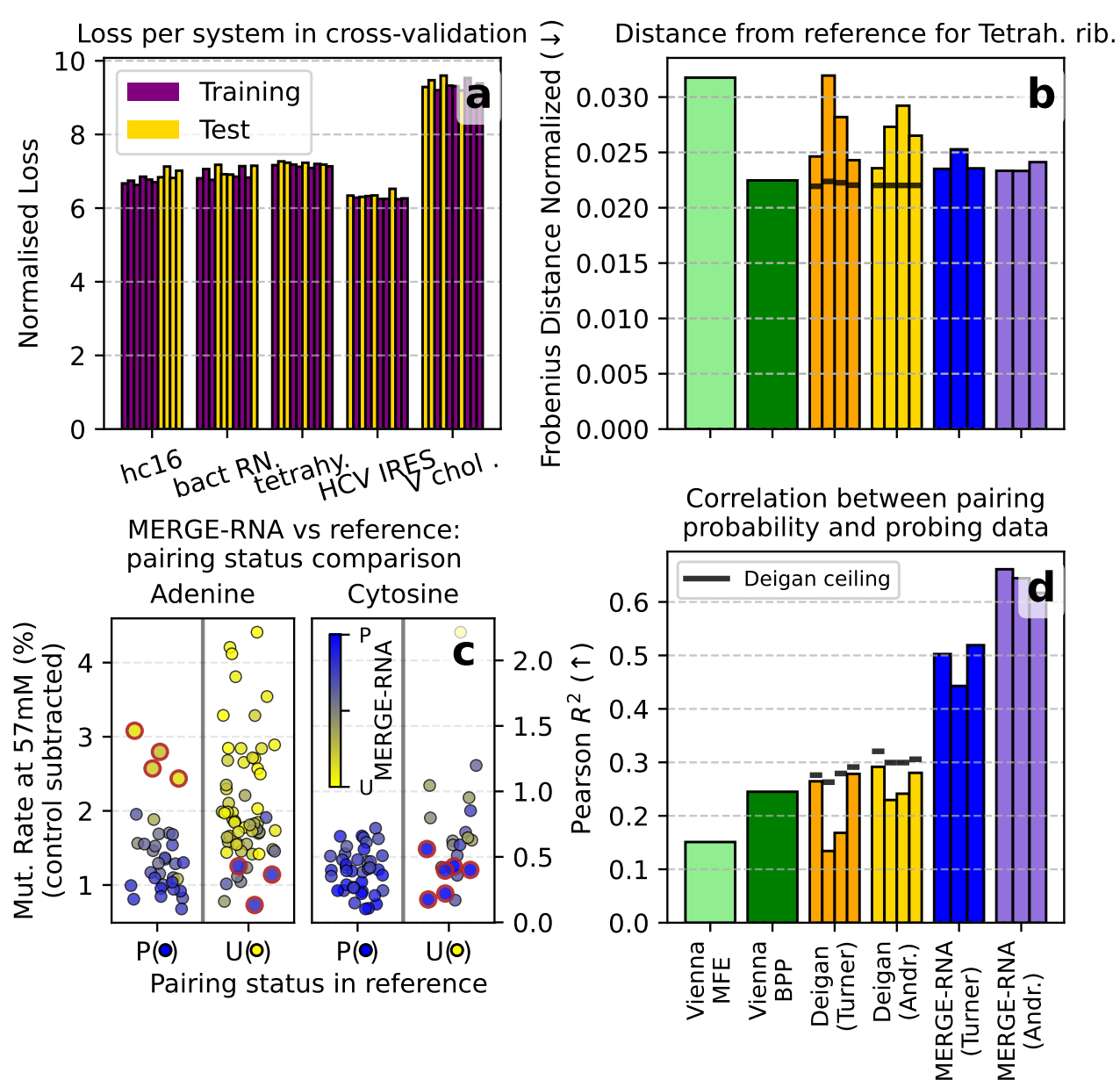

Figure S6 Analysis of *Tetrahymena* ribozyme. This figure is analogous to Fig. 2 in the main text, but here we show results for the *Tetrahymena* ribozyme system.

Training sets	μ_r (kcal/mol)	$\Delta\mu_{\text{pairing}}$	p_{bind}(A, unpaired)	p_{bind}(C, unpaired)	p_{bind}(G)	p_{bind}(U)	m_1
hc16 + HCV + V.chol.	−0.08	0.34	1.00	0.60	0.48	0.05	0.53
hc16 + RNaseP + HCV	−0.05	0.49	1.00	0.57	0.57	0.05	0.50
hc16 + RNaseP + Tetrah.	−0.02	0.15	0.89	0.56	0.28	0.02	0.56
hc16 + RNaseP + V.chol.	−0.36	0.03	1.00	0.66	0.31	0.04	1.05
hc16 + Tetrah. + HCV	−0.06	0.61	0.98	0.44	0.58	0.03	0.50
hc16 + Tetrah. + V.chol.	−0.07	0.40	0.91	0.64	0.40	0.03	0.53
RNaseP + HCV + V.chol.	0.08	0.18	0.99	0.58	0.35	0.05	0.39
RNaseP + Tetrah. + HCV	−0.08	0.24	0.97	0.51	0.35	0.04	0.53
RNaseP + Tetrah. + V.chol.	−0.05	0.79	0.91	0.61	0.41	0.07	0.54
Tetrah. + HCV + V.chol.	−0.09	0.50	1.00	0.49	0.57	0.05	0.45

Table S3. Physical parameters obtained by training MERGE-RNA on different triplets of RNA systems (see section 'Transferability and accuracy of the physical model' in the main text).

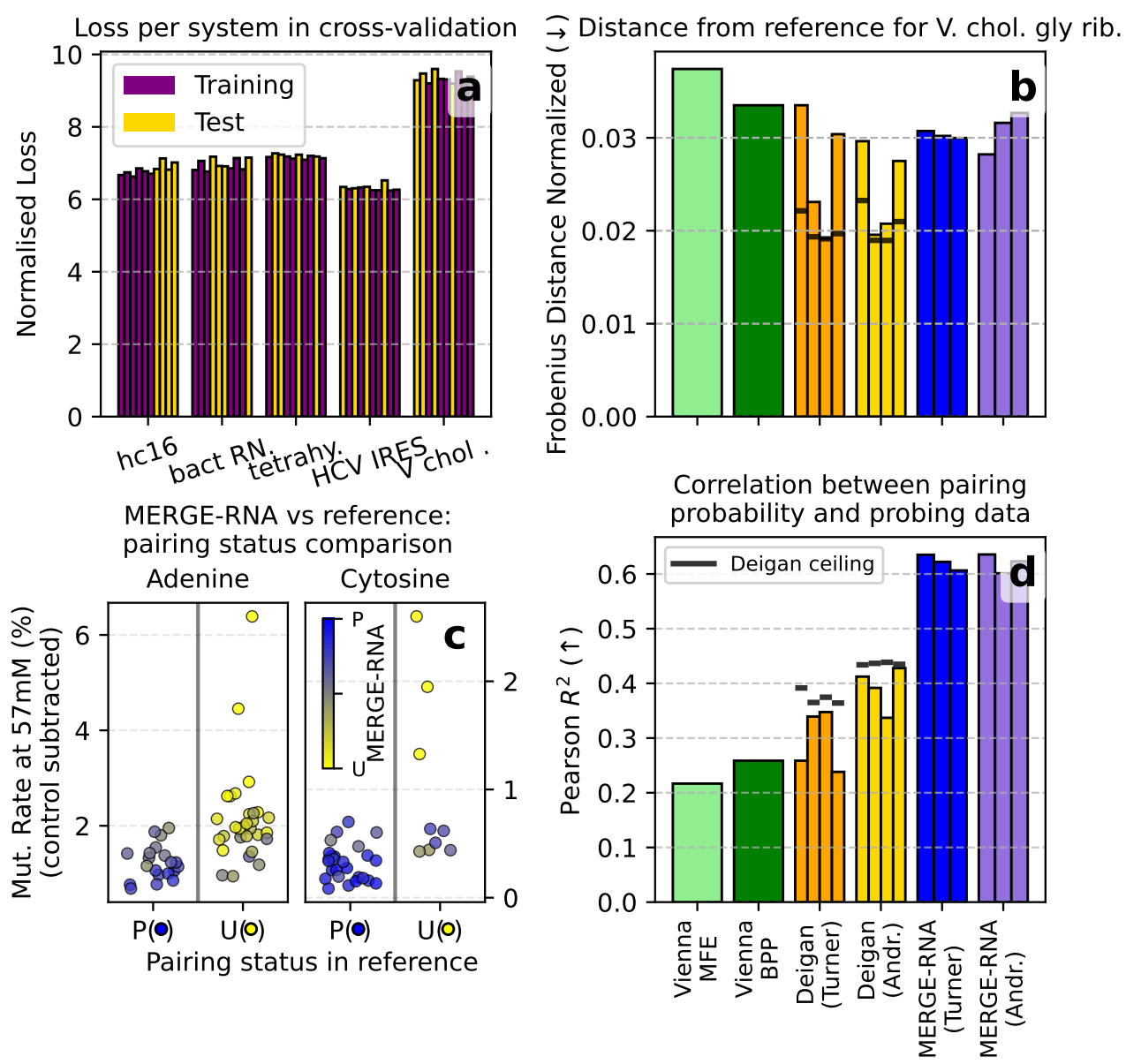

Figure S7 Analysis of *V. cholerae* glycine riboswitch. This figure is analogous to Fig. 2 in the main text, but here we show results for the *V. cholerae* glycine riboswitch system.

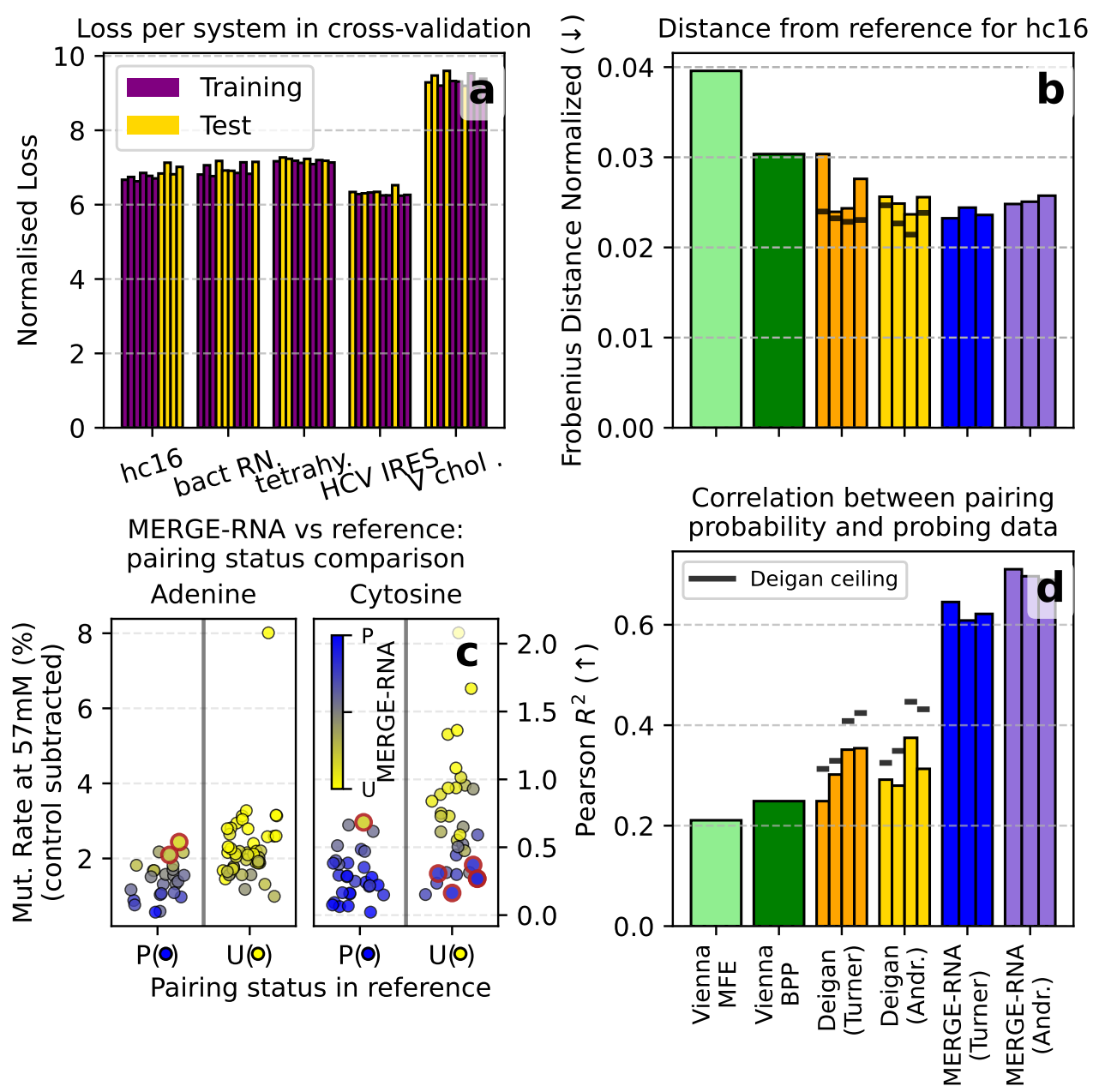

Figure S8 Analysis of HC16. This figure is analogous to Fig. 2 in the main text, but here we show results for the HC16 system.

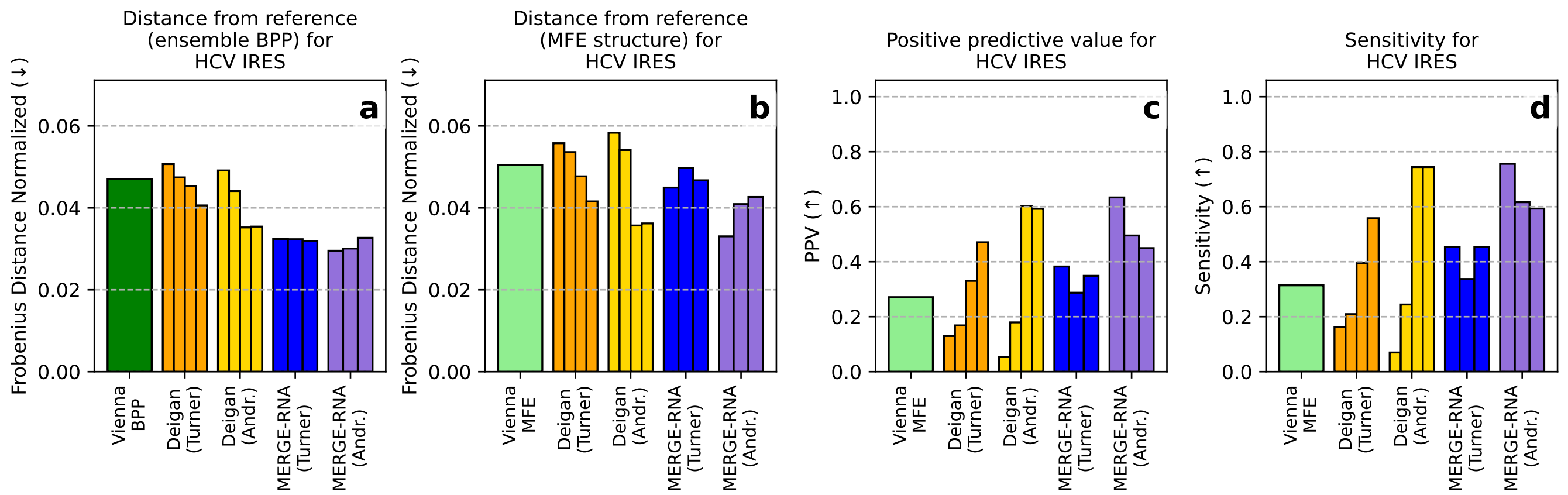

Figure S9 Structure-prediction metrics for the HCV IRES: no-data baseline (ViennaRNA), Deigan pseudo-free-energy approach and MERGE-RNA. **(a)** Frobenius distance to the reference computed on the partition-function ensemble of base-pairing probabilities, as in Fig. 2b; **(b)** the same distance computed on the single minimum-free-energy structure; **(c)** positive predictive value and **(d)** sensitivity of that structure, with ± 1-nt tolerance. Panels (a) and (b) share the vertical scale and differ only in the predicted object, isolating the effect of scoring a single MFE structure. Deigan sub-bars are the four DMS concentrations $[8, 17, 34, 57]$ mM with the leave-one-system-out coefficients; MERGE-RNA sub-bars are three independent fit runs.

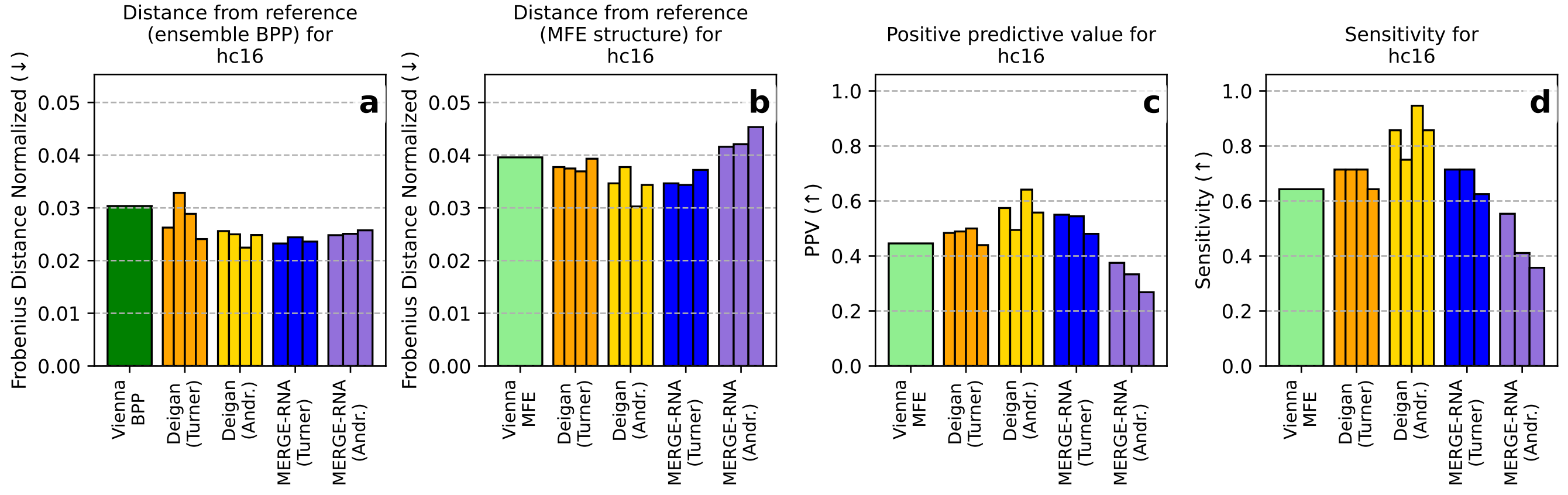

Figure S10 Structure-prediction metrics for the HC16 system, analogous to Fig. S9.

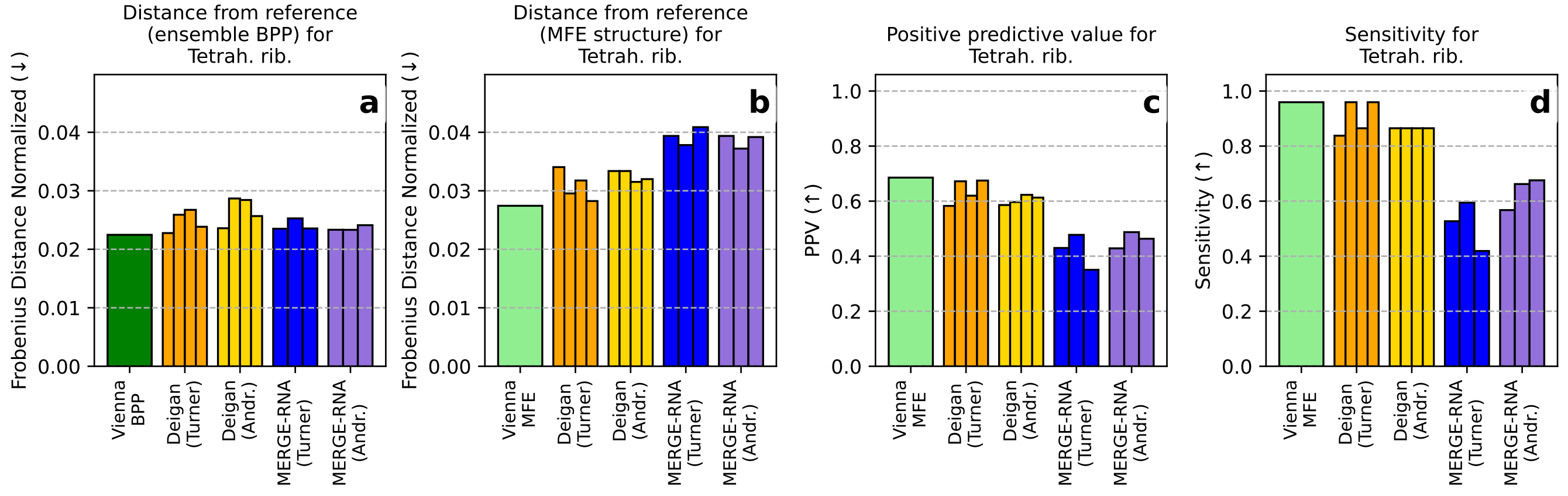

Figure S11 Structure-prediction metrics for the *Tetrahymena* ribozyme, analogous to Fig. S9.

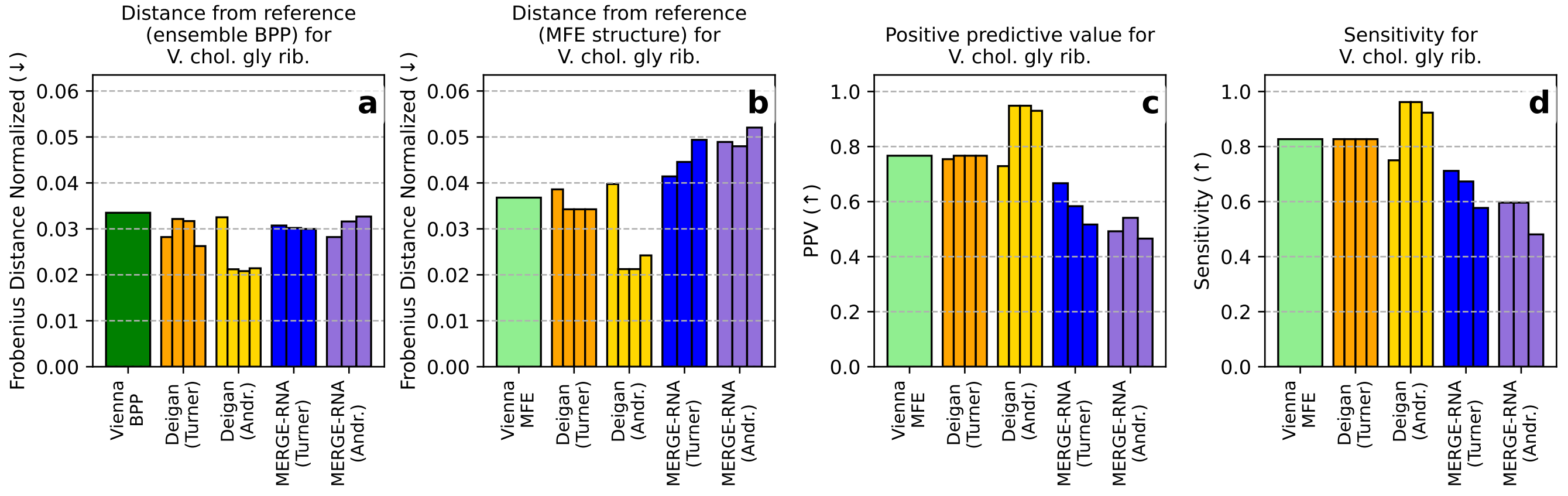

Figure S12 Structure-prediction metrics for the *V. cholerae* glycine riboswitch, analogous to Fig. S9.

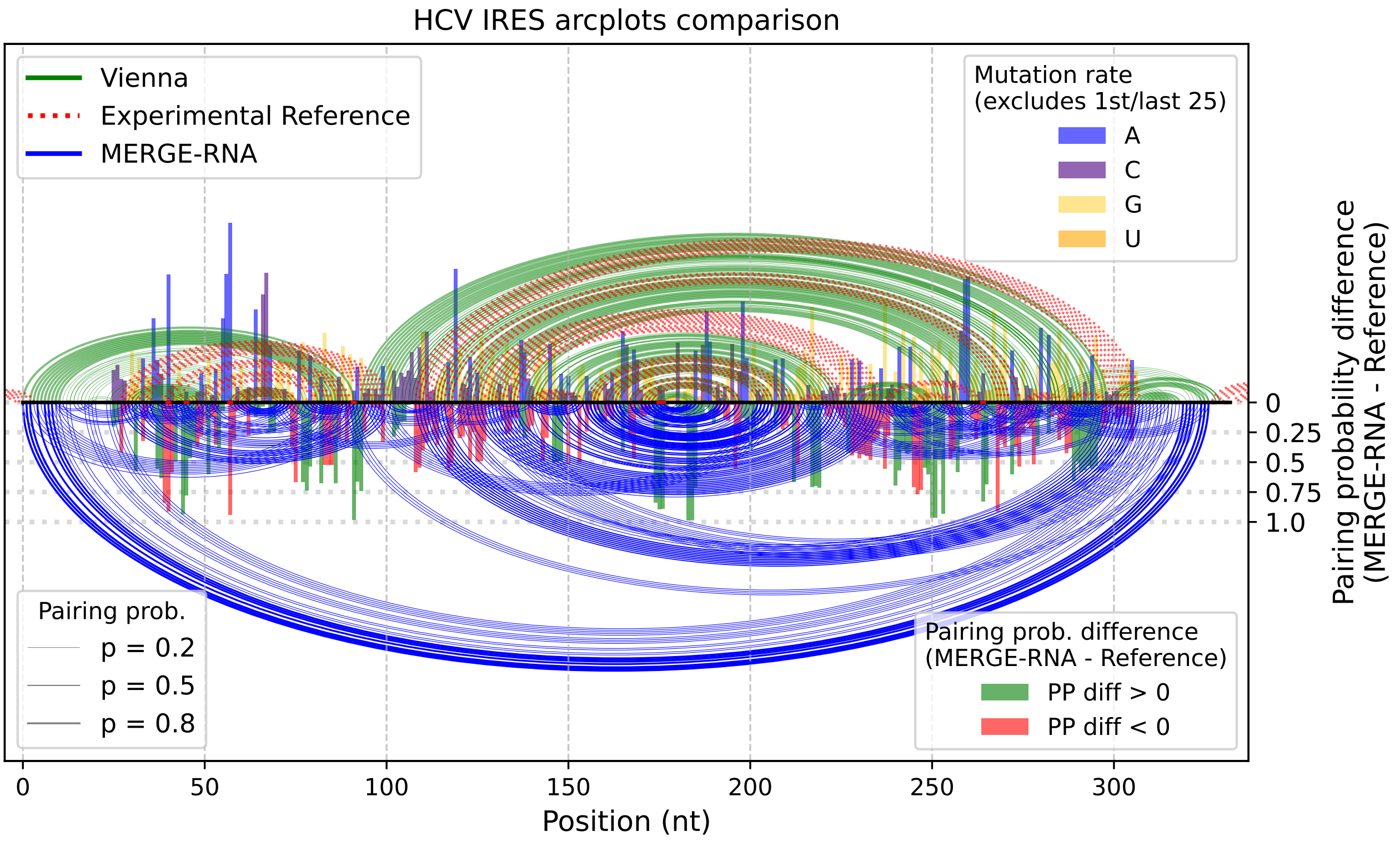

Figure S13 Detailed arc plot representation comparing the secondary structure of the reference structure (red arcs), the baseline thermodynamic ensemble (green arcs) and the MERGE-RNA refined ensemble (blue arcs) for the HCV IRES system. The arc thickness is proportional to the base-pairing probability. Bars above the horizontal line show the experimental mutation rate profiles at 57mM, colored by nucleotide: A (blue), C (violet), G (yellow) and U (orange). First and last 25 nucleotides are not shown since are not used in the model fitting. Bars below the horizontal line show the difference in base-pairing probability between MERGE-RNA and the binary reference structure, colored in green when positive (MERGE-RNA predicts more structure) and red otherwise. 'Strong disagreements' as defined in the main text (where MERGE-RNA predictions are $< 25\%$ or $> 75\%$ while disagreeing with the reference structure for A and C nucleotides) are highlighted in red on the horizontal axis.

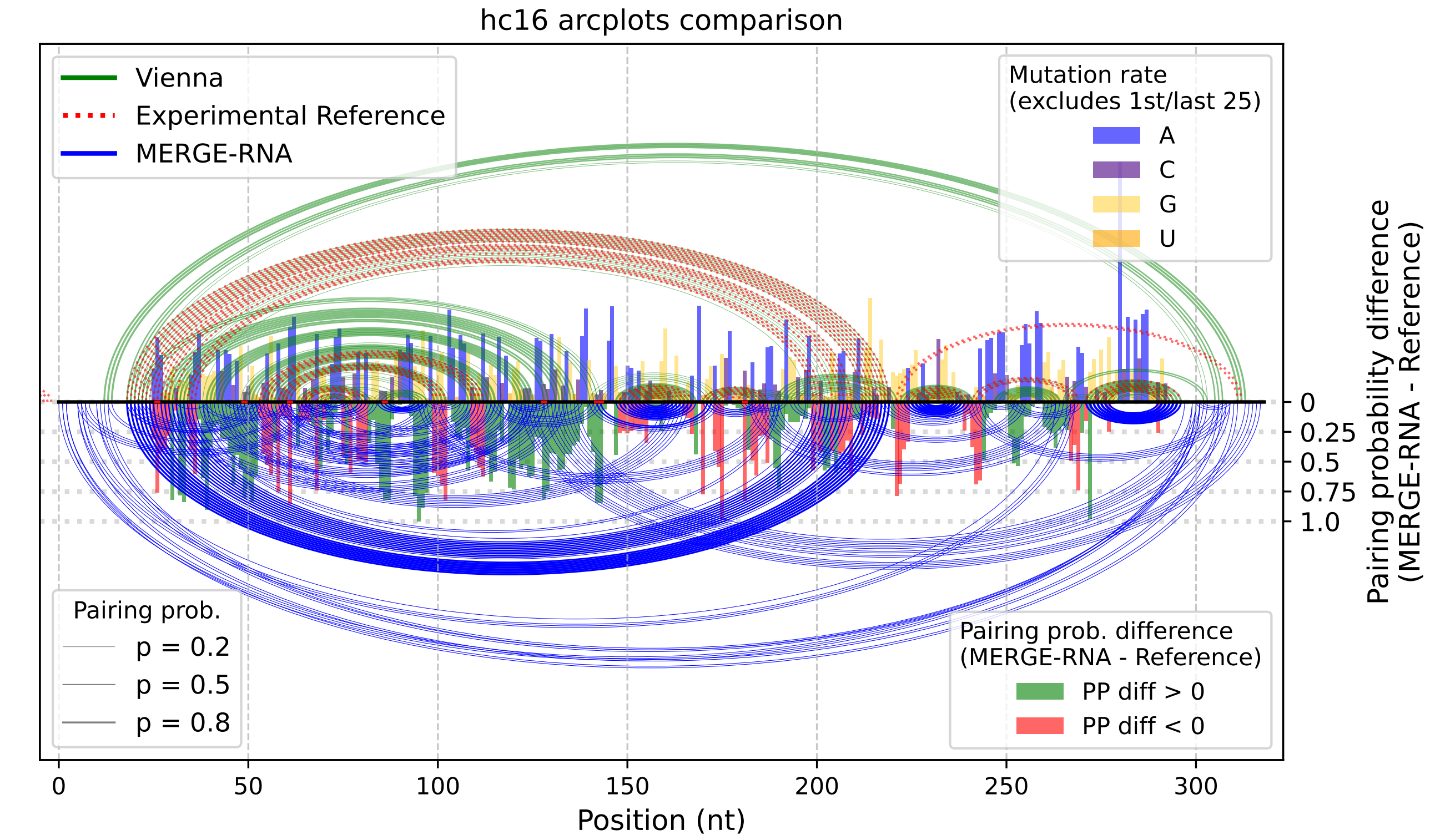

Figure S14 Detailed arc plot representation for the HC16 system, analogous to Fig. S13.

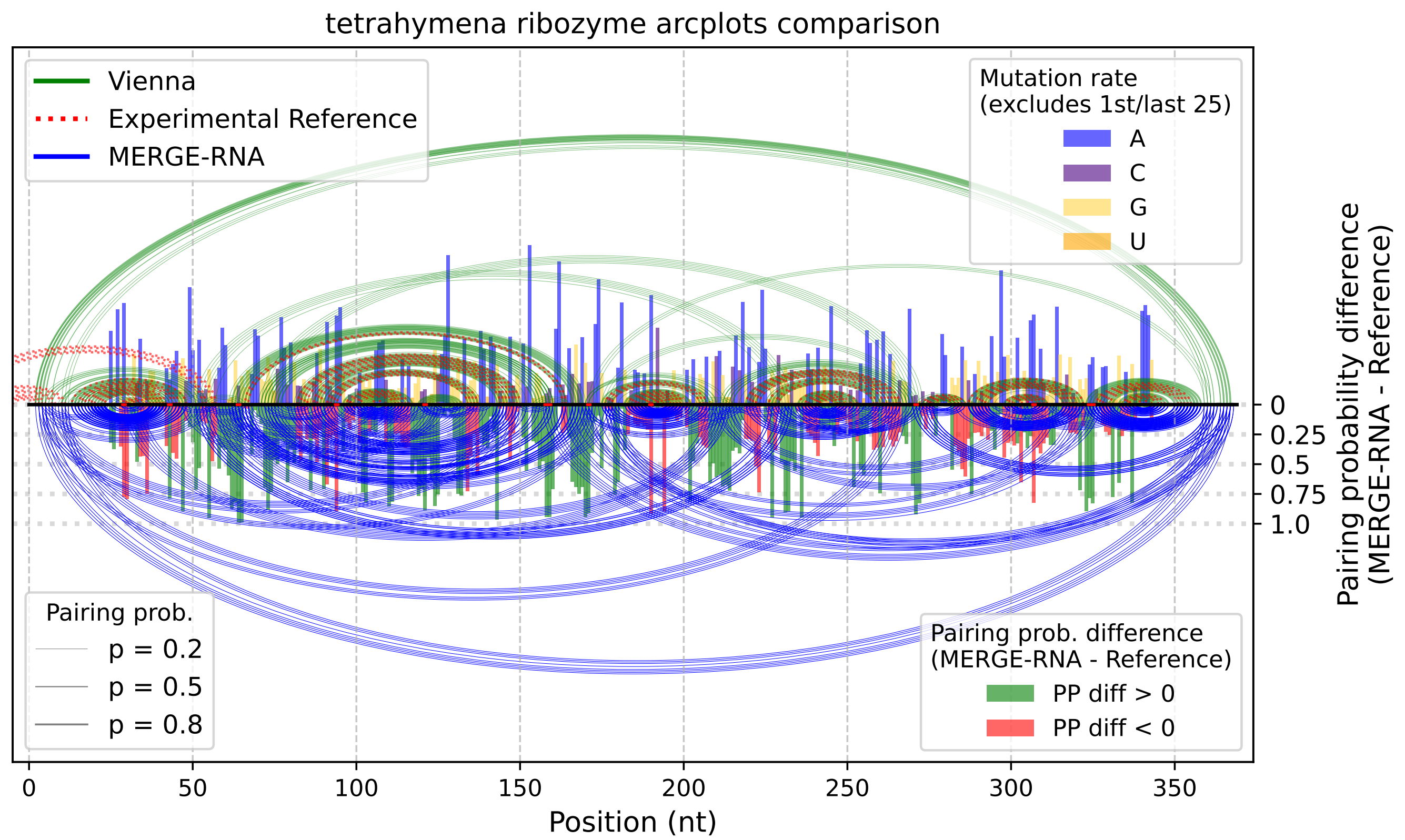

Figure S15 Detailed arc plot representation for the *Tetrahymena* ribozyme system, analogous to Fig. S13.

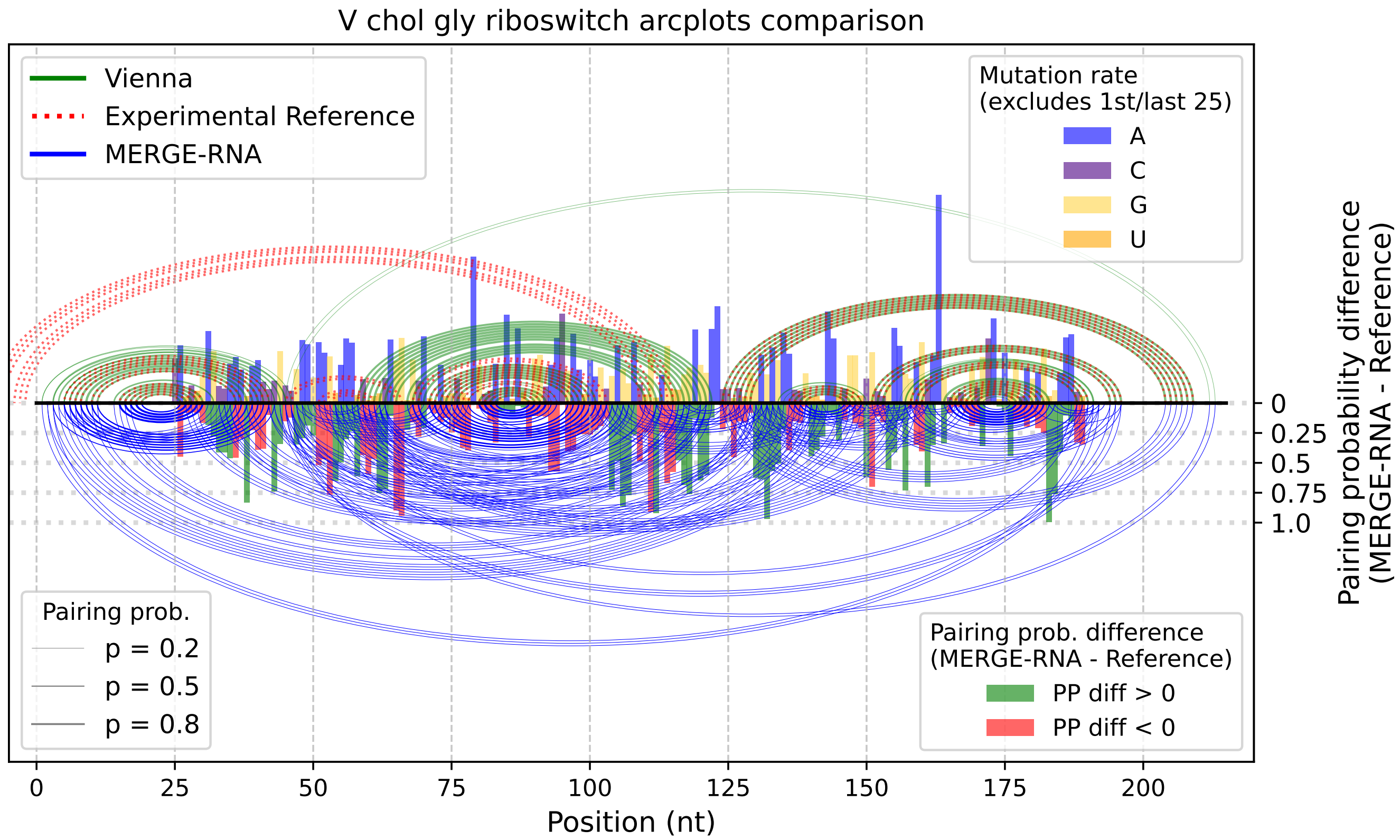

Figure S16 Detailed arc plot representation for the *V. cholerae* glycine riboswitch system, analogous to Fig. S13.

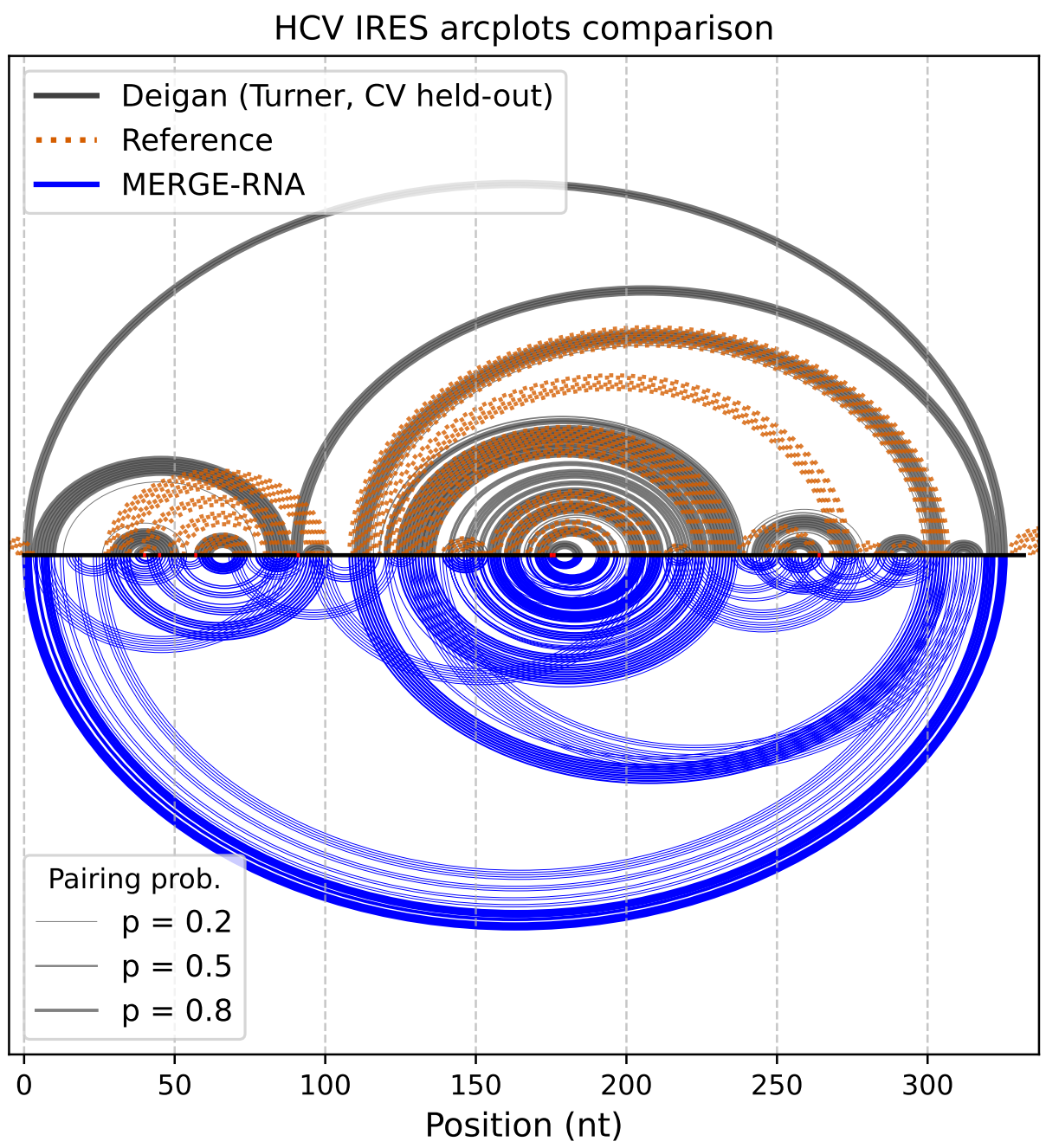

Figure S17 Arc plot for the HCV IRES, as Fig. 3 of the main text but with the upper solid arcs (dark grey) showing the Deigan ensemble instead of the unconstrained thermodynamic one, computed at 57 mM under the Turner 2004 parameters with the leave-one-system-out coefficients of Table S2. Reference (red dotted) and MERGE-RNA (blue) are unchanged, as are the strong-disagreement marks on the axis, which are computed between MERGE-RNA and the reference.

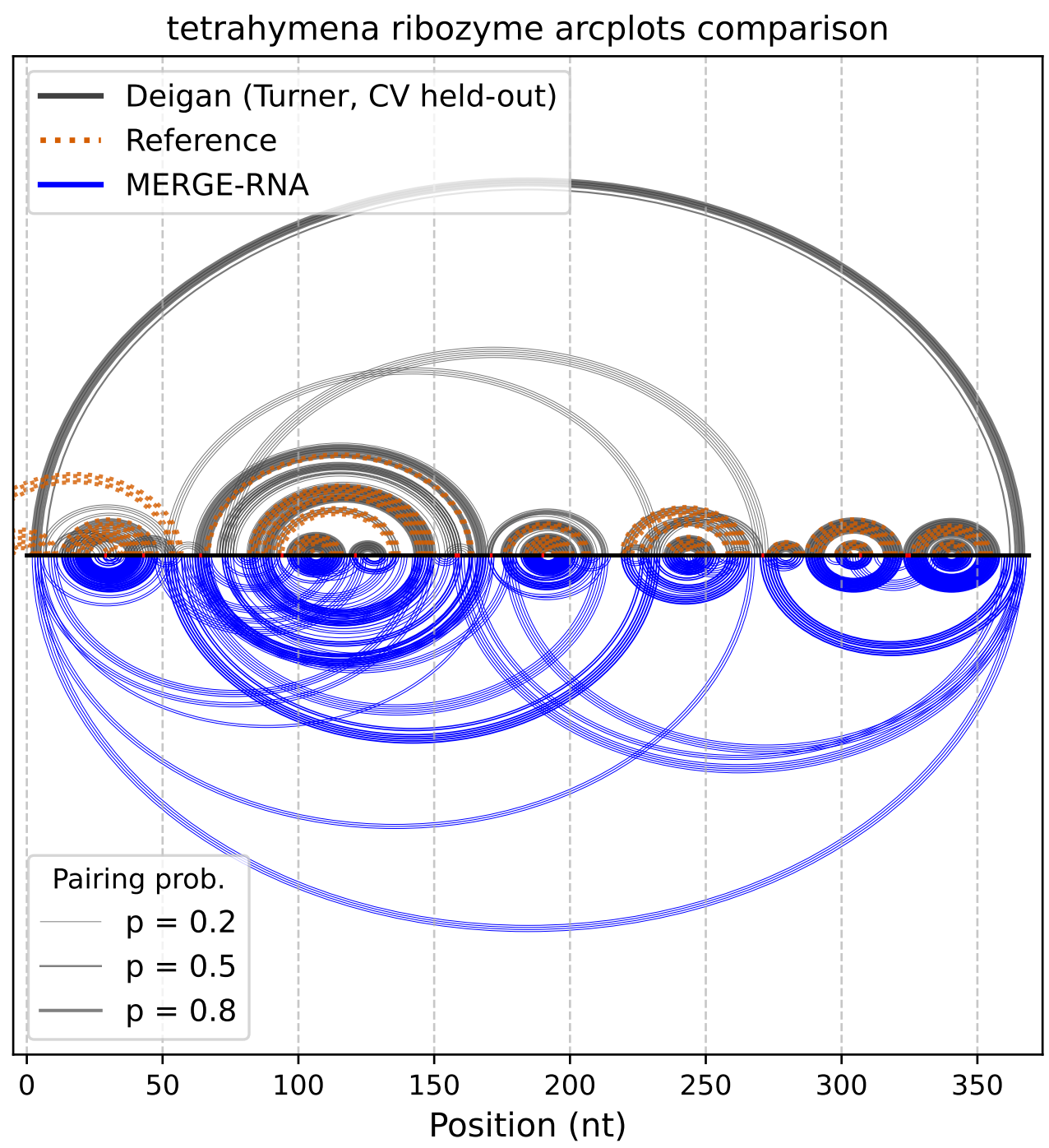

Figure S19 Arc plot representation with the Deigan ensemble for the *Tetrahymena* ribozyme system, analogous to Fig. S17.

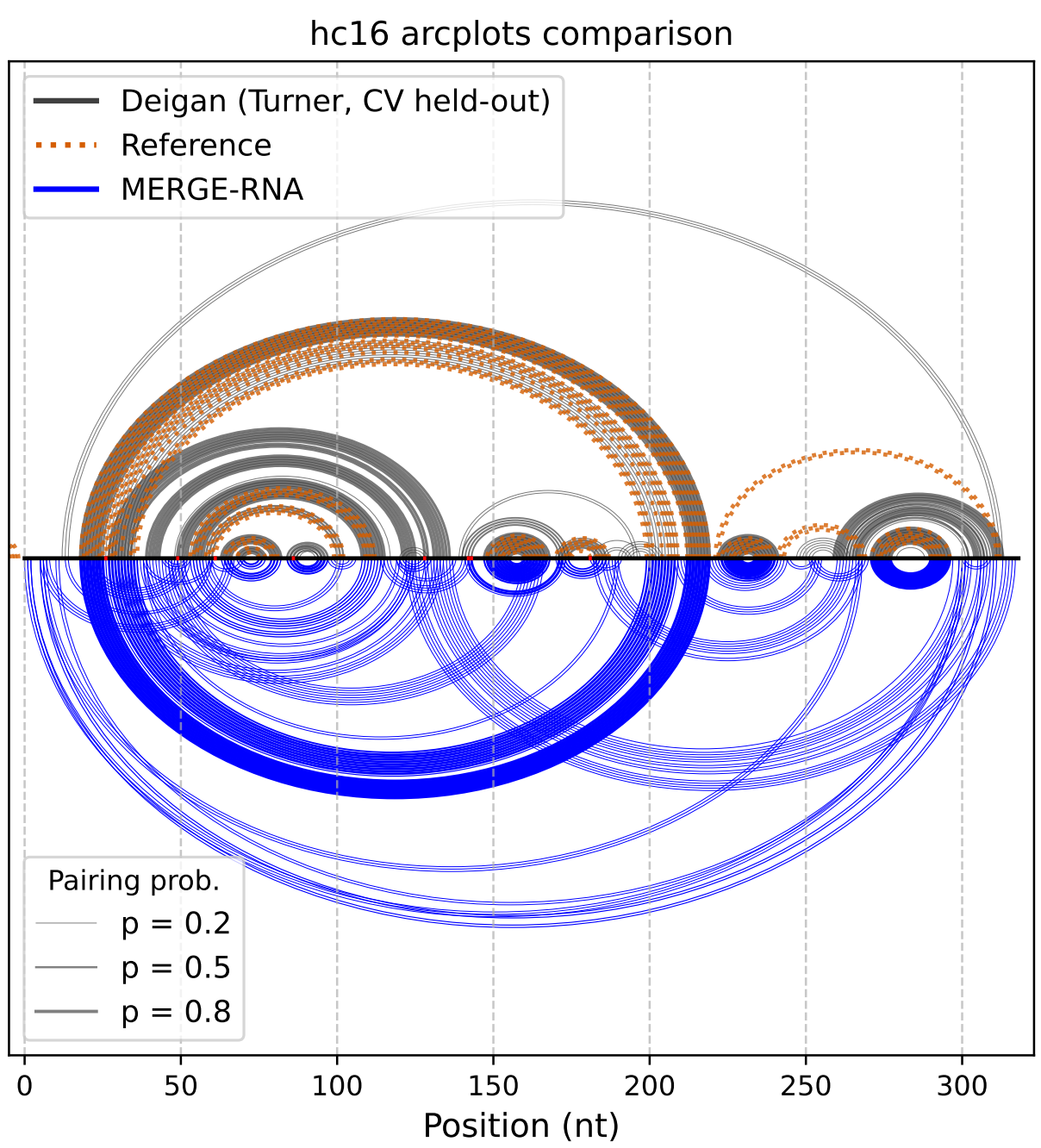

Figure S18 Arc plot representation with the Deigan ensemble for the HC16 system, analogous to Fig. S17.

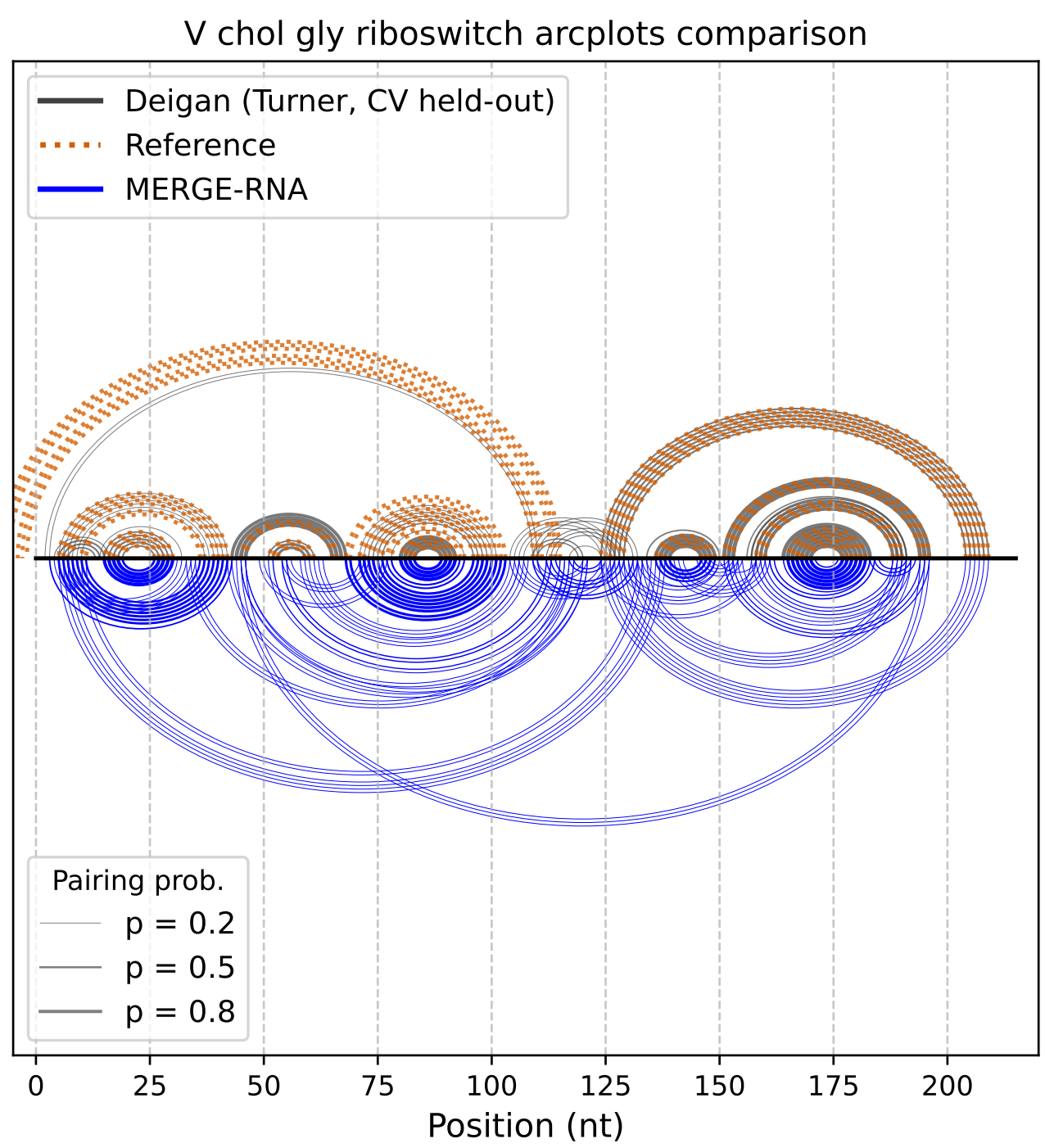

Figure S20 Arc plot representation with the Deigan ensemble for the *V. cholerae* glycine riboswitch system, analogous to Fig. S17.

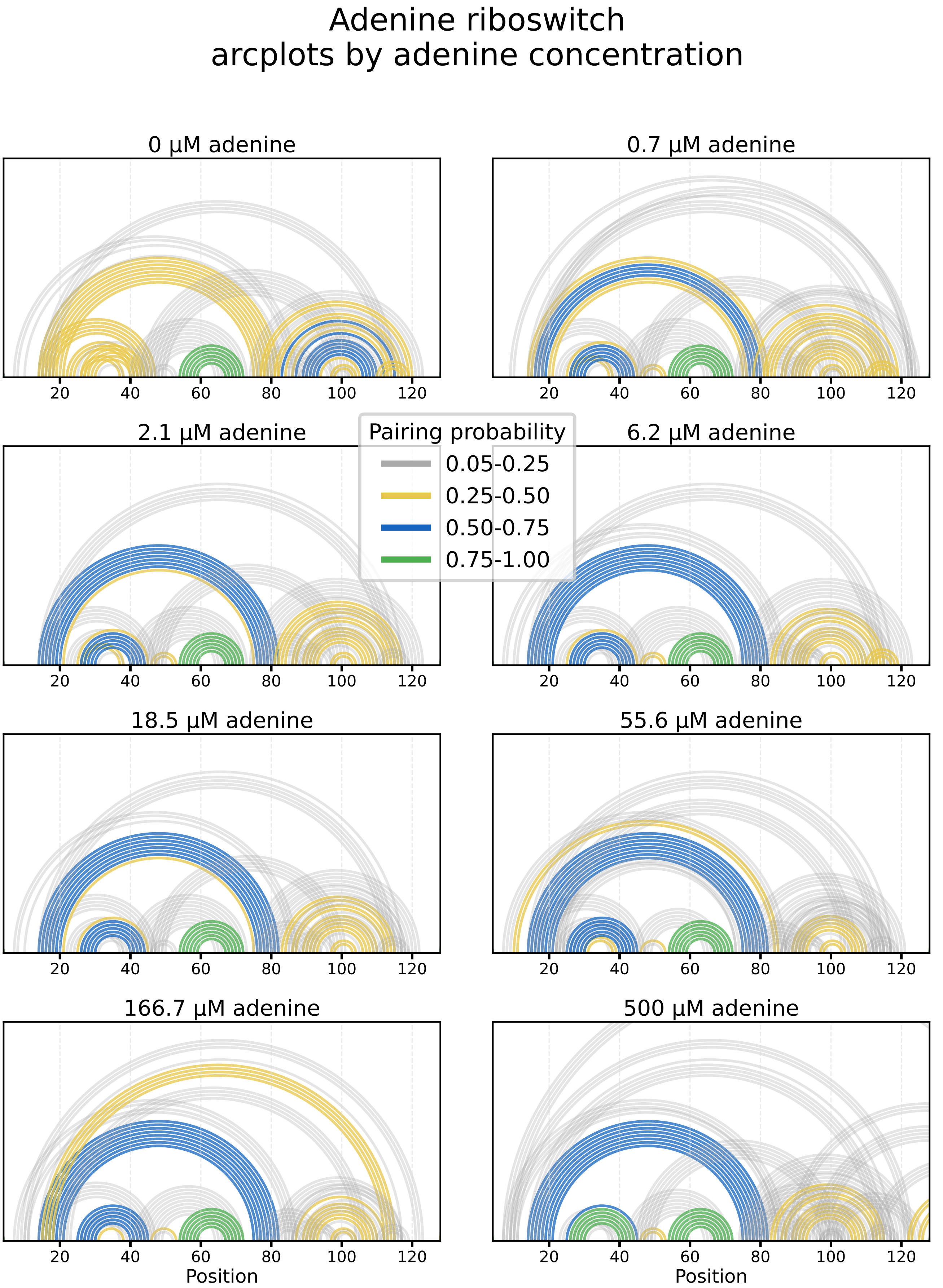

Figure S21 Arc plot representation for the MERGE-RNA inferred pairing probabilities for the *V. vulnificus* adenine riboswitch, when trained on data collected at different ligand concentrations. Colors indicate the base-pairing probability.

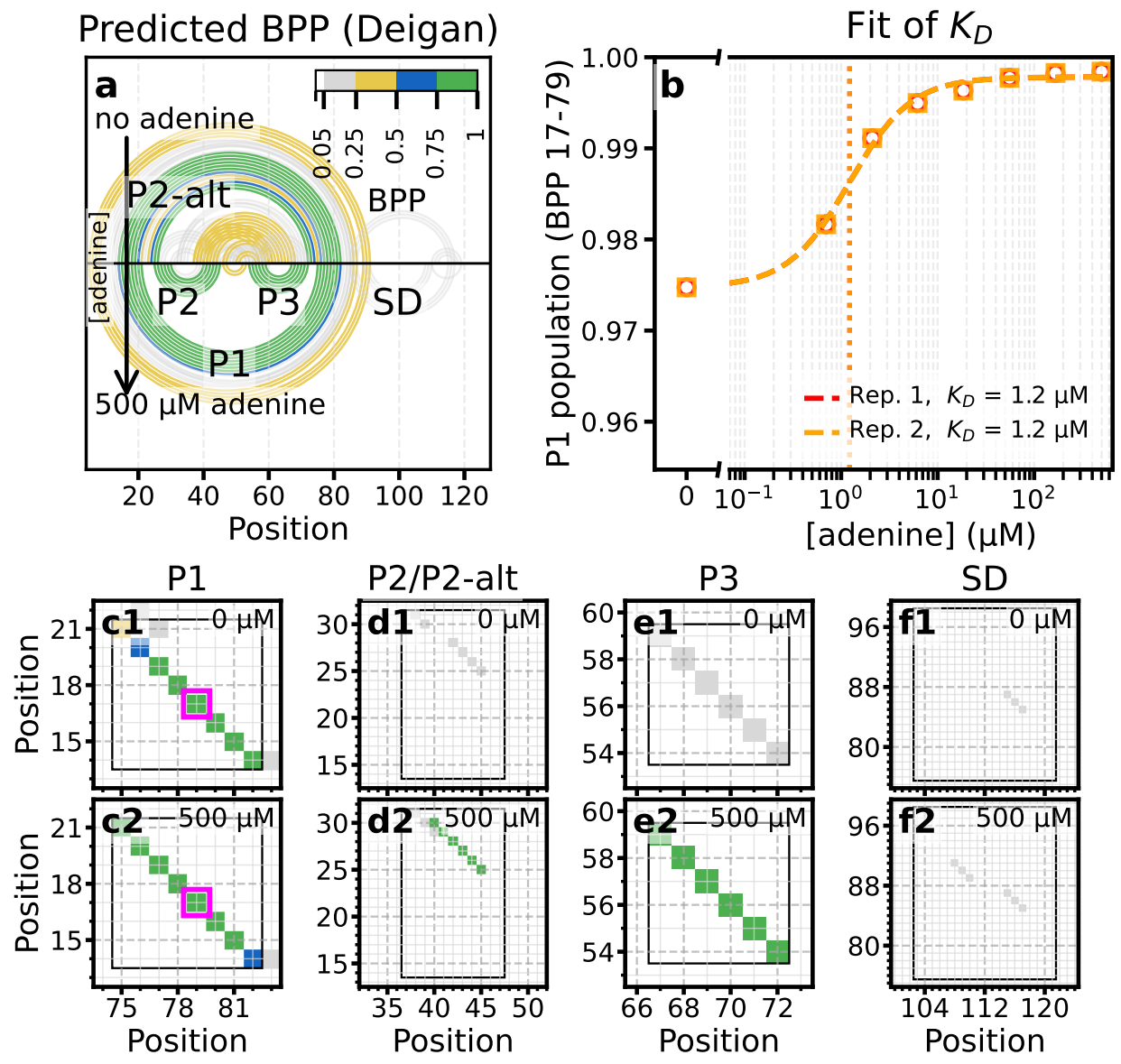

Figure S22 Same analysis as in Fig. 4 of the main text, for the *V. vulnificus* adenine riboswitch, but obtained with the Deigan pseudo-free-energy approach (Refitting the Deigan slope and intercept) instead of MERGE-RNA.

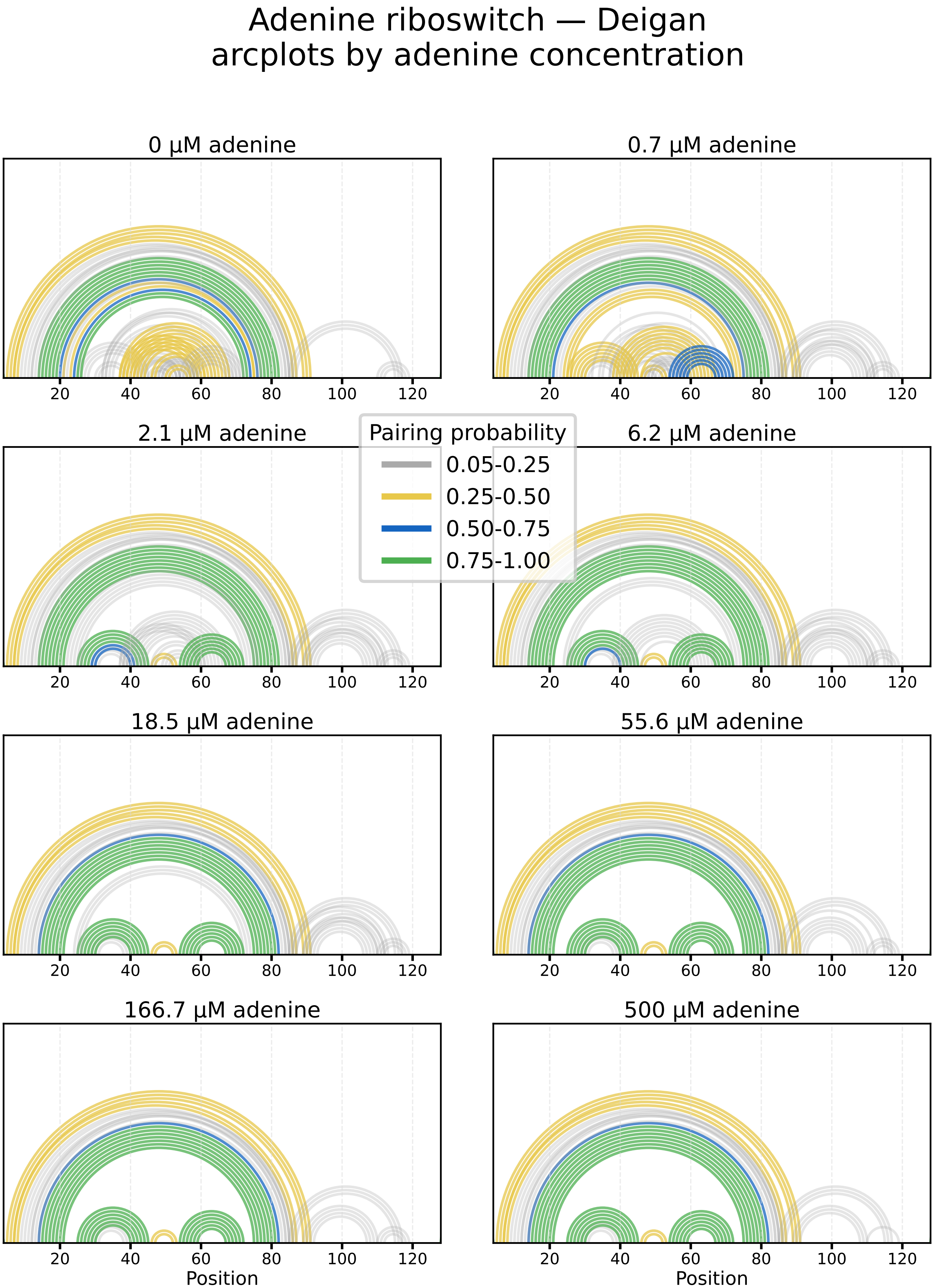

Figure S23 Same analysis as in Fig. S21, but obtained with the Deigan pseudo-free-energy approach. Colors indicate the base-pairing probability.

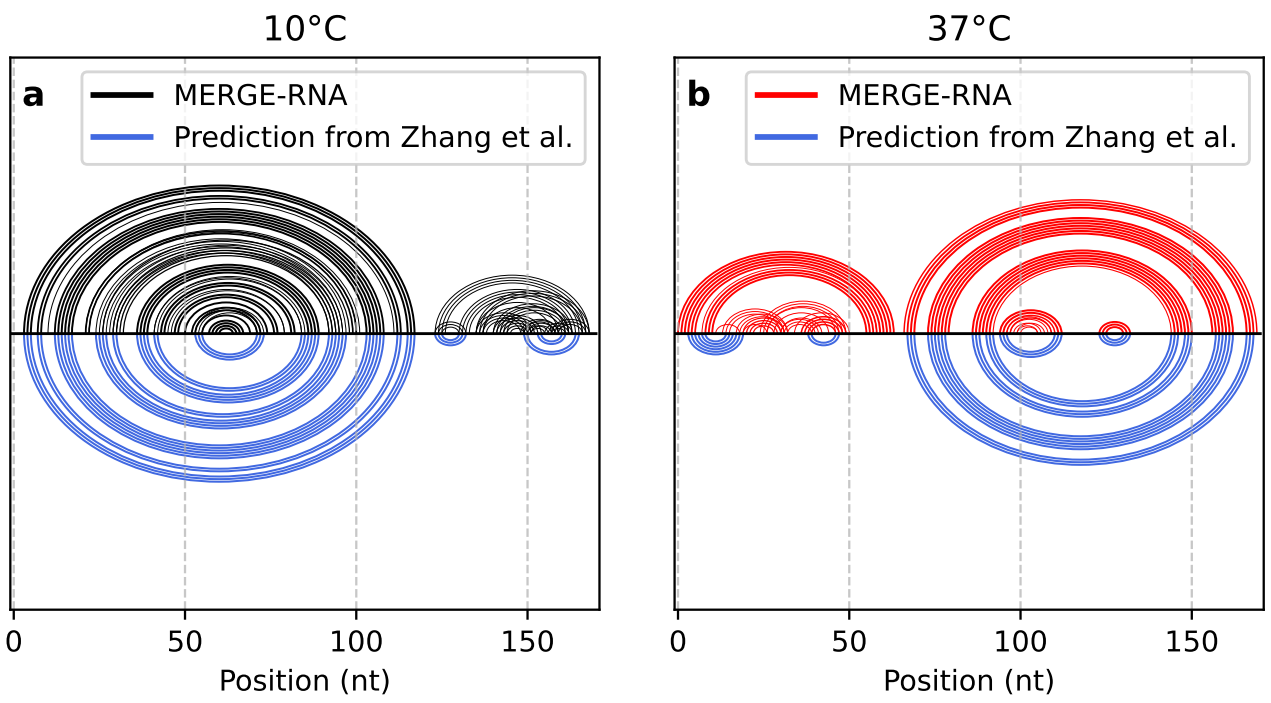

Figure S24 Arc plot visualization of the *cspA* $5'$ UTR showing MERGE-RNA ensembles at 10°C (black) and 37°C (red), compared to the single structure prediction from ref. (42). This figure is analogous to Fig. S2, but shows results from a different random initialization of the fitting parameters (the main text presents the solution with lowest loss).

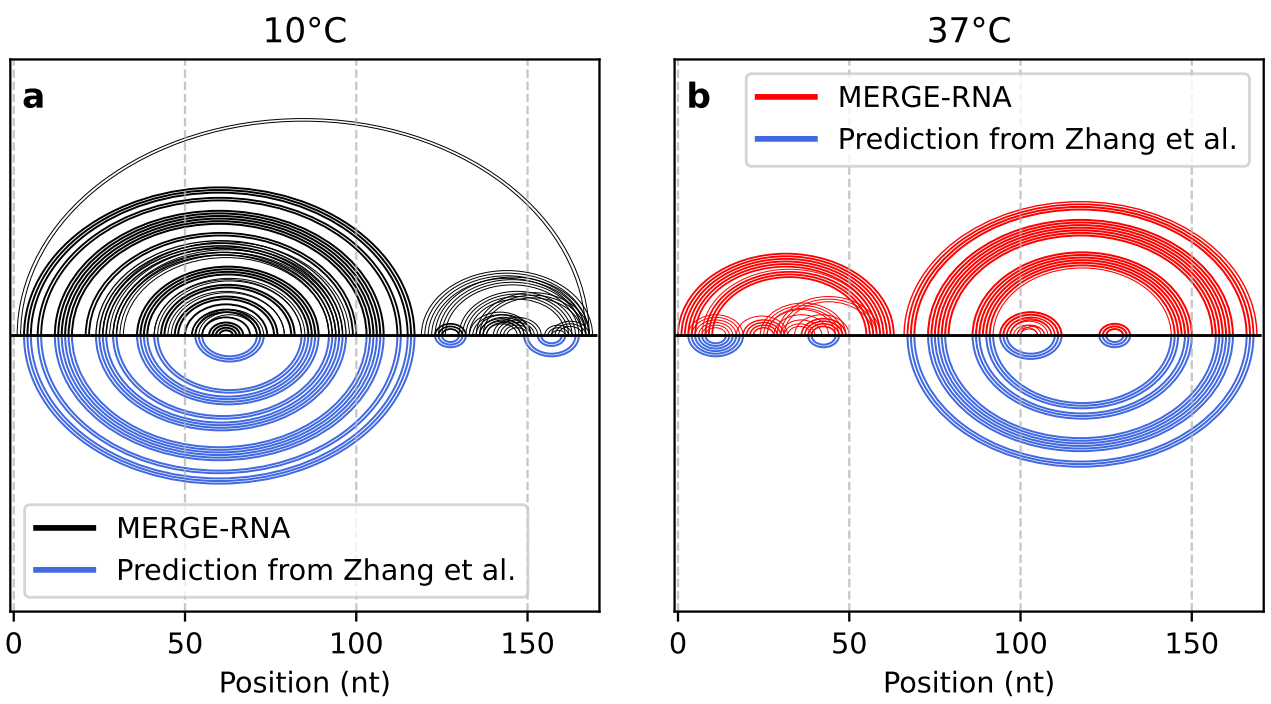

Figure S25 Arc plot visualization of the *cspA* $5'$ UTR showing MERGE-RNA ensembles at 10°C (black) and 37°C (red), compared to the single structure prediction from ref. (42). This figure is analogous to Fig. S2, but shows results from a different random initialization of the fitting parameters (the main text presents the solution with lowest loss).

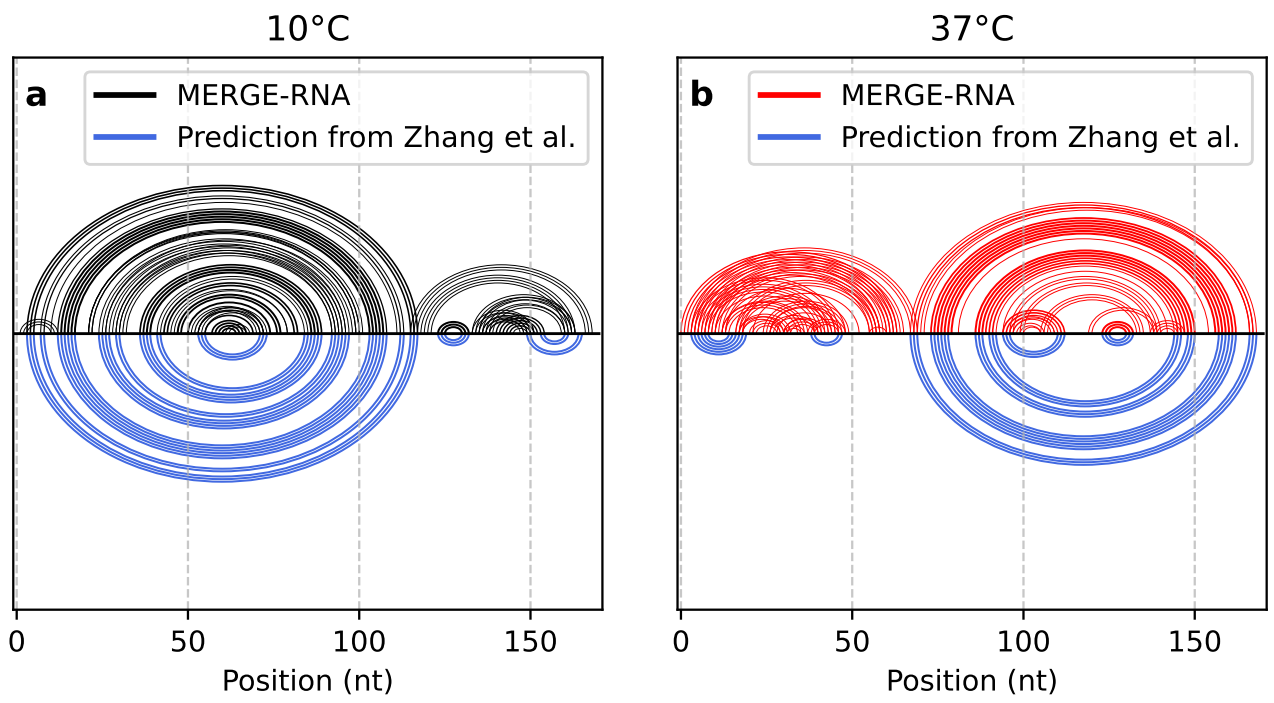

Figure S26 Arc plot visualization of the *cspA* $5'$ UTR showing MERGE-RNA ensembles at 10°C (black) and 37°C (red), compared to the single structure prediction from ref. (42). This figure is analogous to Fig. S2, but shows results from a different random initialization of the fitting parameters (the main text presents the solution with lowest loss).

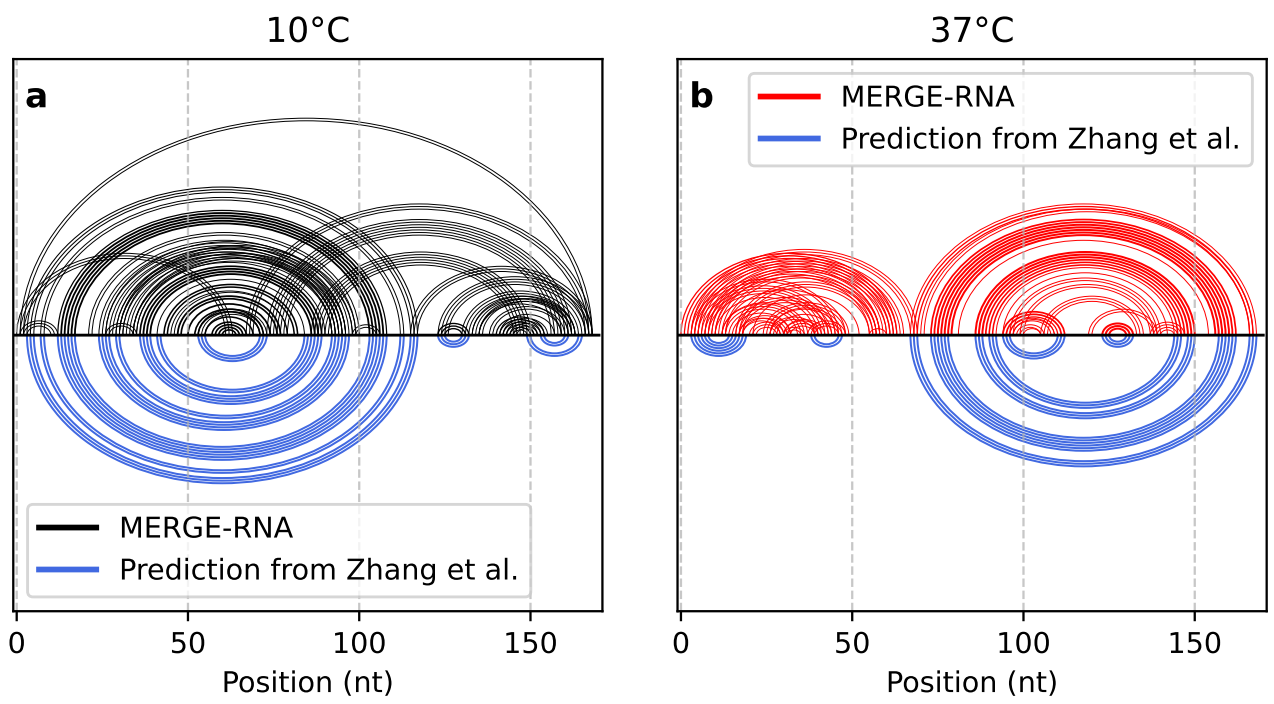

Figure S27 Arc plot visualization of the *cspA* $5'$ UTR showing MERGE-RNA ensembles at 10°C (black) and 37°C (red), compared to the single structure prediction from ref. (42). This figure is analogous to Fig. S2, but shows results from a different random initialization of the fitting parameters (the main text presents the solution with lowest loss).

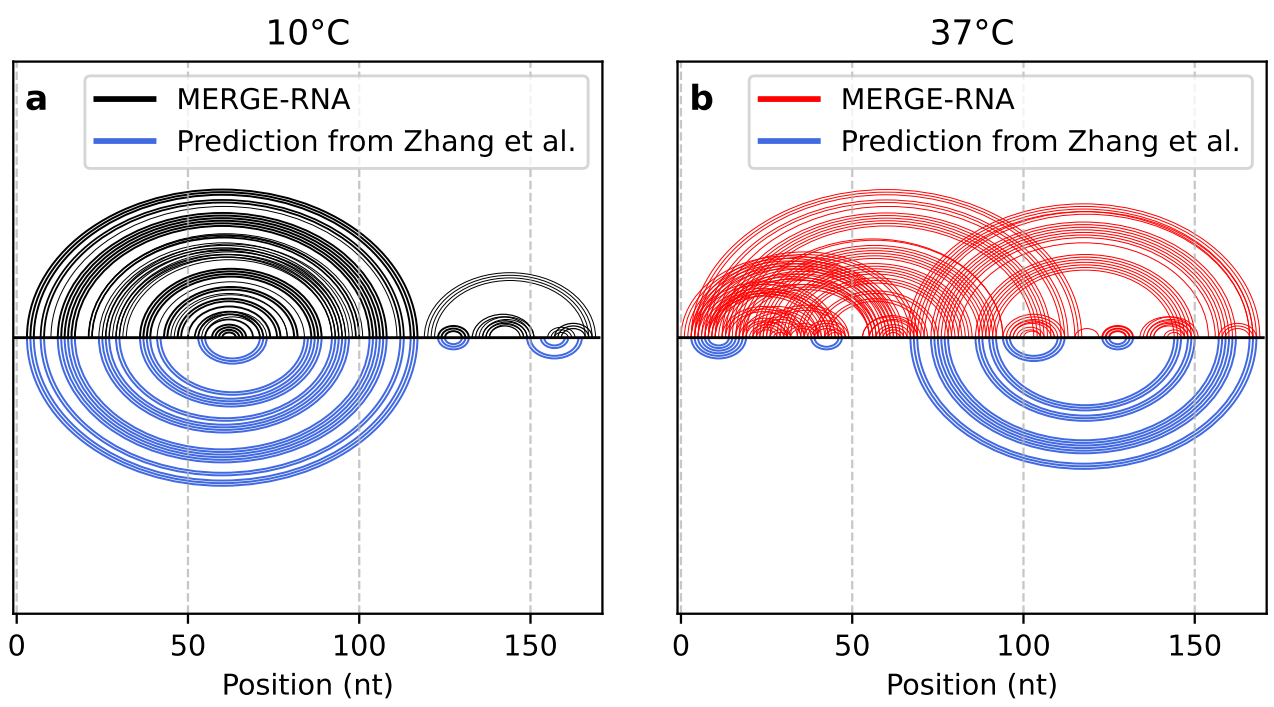

Figure S28 Arc plot visualization of the *cspA* $5'$ UTR showing MERGE-RNA ensembles at 10°C (black) and 37°C (red), compared to the single structure prediction from ref. (42). This figure is analogous to Fig. S2, but shows results from a different random initialization of the fitting parameters (the main text presents the solution with lowest loss).

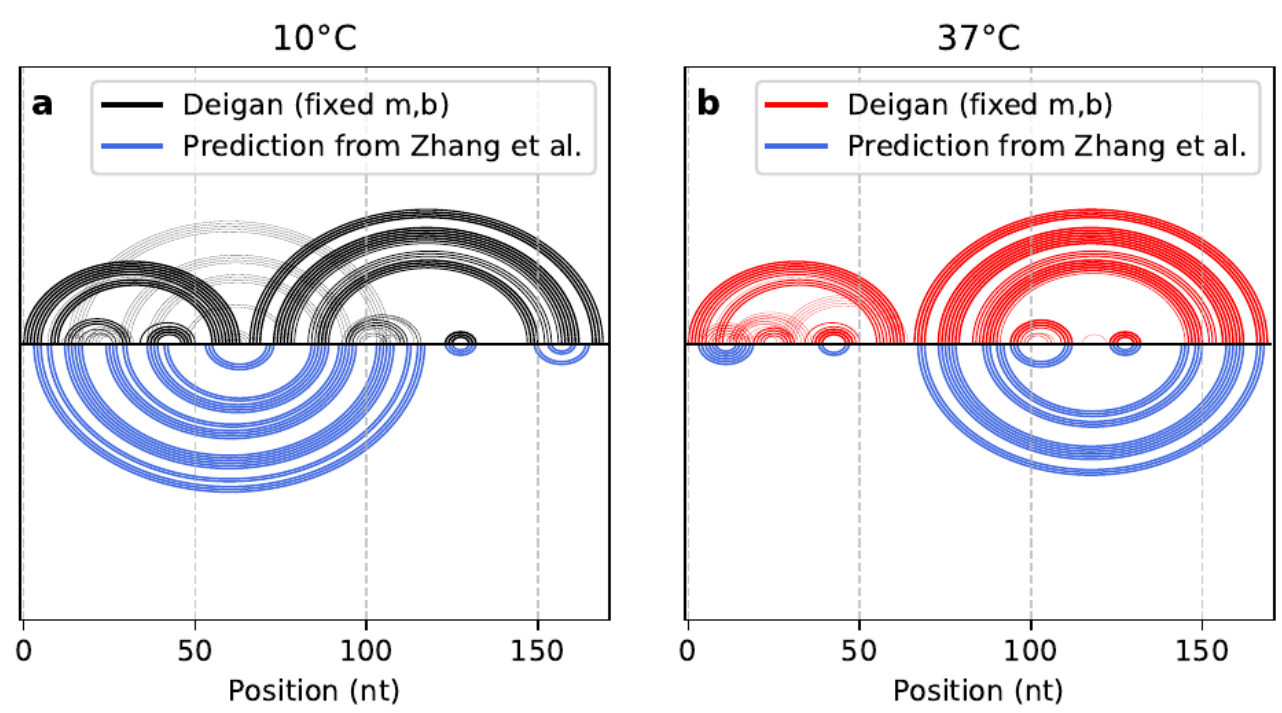

Figure S29 Same analysis as in Fig. S2, but obtained with the Deigan pseudo-free-energy approach (fixed m, b) instead of MERGE-RNA. **(a,b)** Arc plots for the DMS data collected at 10 and 37°C, respectively. The Deigan predictions (black and red) are compared with the single-structure prediction of Ref. (42) (blue).

Table S4. Loop co-occupancy inferred from baseline thermodynamic ensembles and after MERGE-RNA refinement for the putative bistable sequence (see section 'a putatively bistable sequence exhibits a heterogeneous ensemble with strand displacement'). Entries report the percentage of structures in which Loop1 and Loop2 are formed, using either the Turner2004 (36) or Andronescu2007 (38) baseline parameter sets.

	Baseline			MERGE-RNA		
Turner		Loop1			Loop1	
	Loop2	0	1	Loop2	0	1
	0	0%	49%	0	0%	27%
	1	51%	0%	1	16%	57%
Andr.		Loop1			Loop1	
	Loop2	0	1	Loop2	0	1
	0	0%	19%	0	0%	42%
	1	81%	0%	1	16%	42%

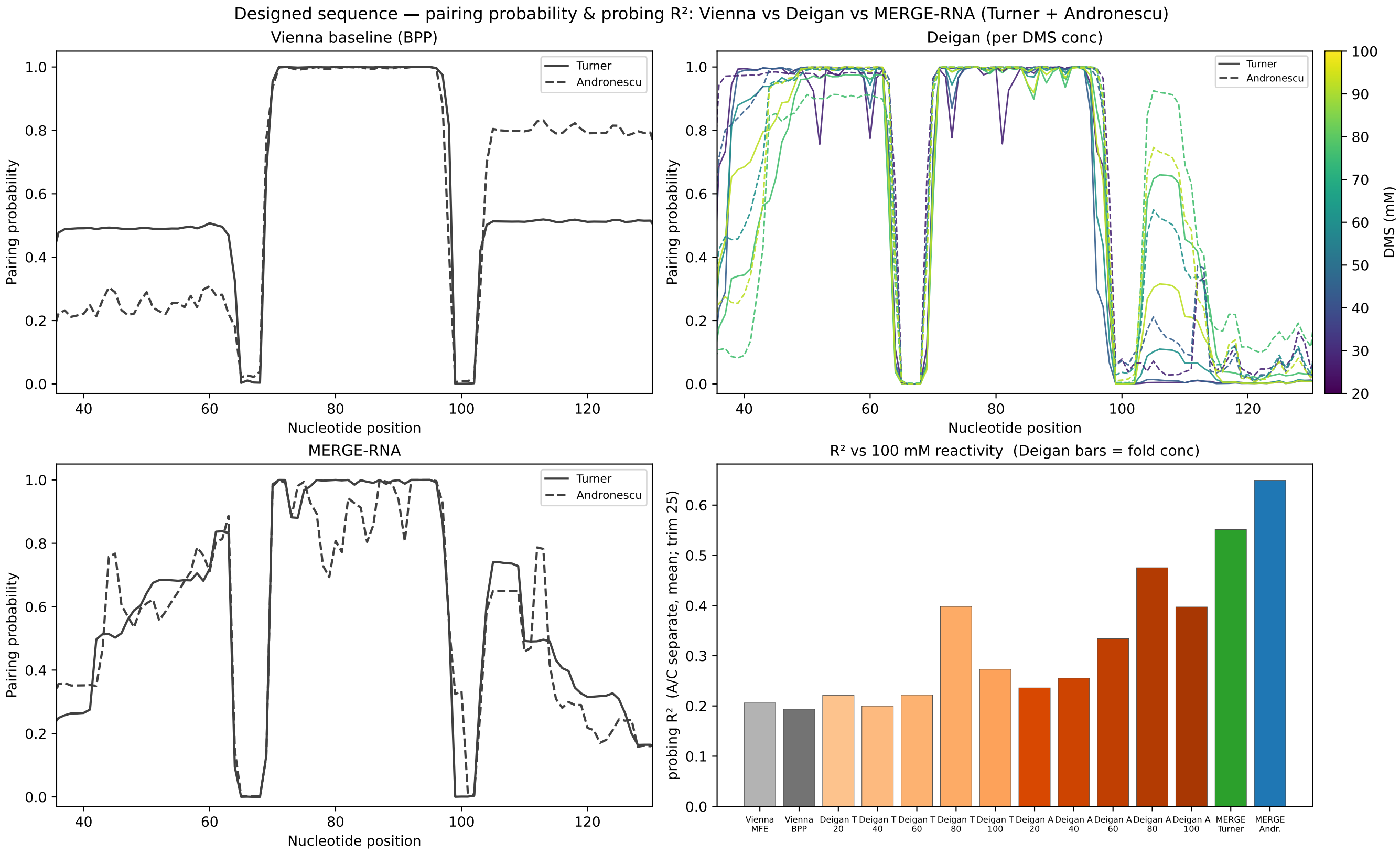

Figure S30 Same analysis as in Fig. 6, for the designed sequence, comparing the Vienna baseline, the Deigan pseudo-free-energy approach (per DMS concentration), and MERGE-RNA under the Turner 2004 and Andronescu 2007 parameters. The bottom-right panel reports the probing R^2 against the 100 mM reactivity profile.
